# Polymer Uncrossing and Knotting in Protein Folding, and Their Role in Minimal Folding Pathways

**DOI:** 10.1371/journal.pone.0053642

**Published:** 2013-01-24

**Authors:** Ali R. Mohazab, Steven S. Plotkin

**Affiliations:** Department of Physics and Astronomy, University of British Columbia, Vancouver, B.C, Canada; Weizmann Institute of Science, Israel

## Abstract

We introduce a method for calculating the extent to which chain non-crossing is important in the most efficient, optimal trajectories or pathways for a protein to fold. This involves recording all unphysical crossing events of a ghost chain, and calculating the minimal uncrossing cost that would have been required to avoid such events. A depth-first tree search algorithm is applied to find minimal transformations to fold 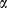, 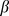, 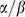, and knotted proteins. In all cases, the extra uncrossing/non-crossing distance is a small fraction of the total distance travelled by a ghost chain. Different structural classes may be distinguished by the amount of extra uncrossing distance, and the effectiveness of such discrimination is compared with other order parameters. It was seen that non-crossing distance over chain length provided the best discrimination between structural and kinetic classes. The scaling of non-crossing distance with chain length implies an inevitable crossover to entanglement-dominated folding mechanisms for sufficiently long chains. We further quantify the minimal folding pathways by collecting the sequence of uncrossing moves, which generally involve leg, loop, and elbow-like uncrossing moves, and rendering the collection of these moves over the unfolded ensemble as a multiple-transformation “alignment”. The consensus minimal pathway is constructed and shown schematically for representative cases of an 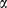, 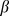, and knotted protein. An overlap parameter is defined between pathways; we find that 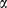 proteins have minimal overlap indicating diverse folding pathways, knotted proteins are highly constrained to follow a dominant pathway, and 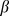 proteins are somewhere in between. Thus we have shown how topological chain constraints can induce dominant pathway mechanisms in protein folding.

## Introduction

Protein folding is a structural transformation, from a disordered-polymer conformational ensemble to an ordered, well-defined structure. Quantifying the dynamical mechanism by which this occurs has been a long-standing problem of interest to both theorists and experimentalists [1]–[16]. It is currently not possible experimentally to capture the full dynamical mechanism of a folding protein in atomic detail, start to finish. Photon counting analyses of single molecule folding trajectories can now extract the mean transition path time across the distribution of productive folding pathways [17]. Typically however, snapshots of the participation of various residues in the folding transition state are used to infer the relative importance of amino acids in defining the protein folding nucleus [6], [18]–[31]. An idea of how the nucleus grows as folding proceeds may be gained by exploring the native shift in the transition state as denaturant concentration is increased [32], but ideally the goal is to quantify folding mechanisms under constant environmental conditions. To this end, simulations and theory have proved an invaluable tool [12], [13], [33]–[43], and have in many respects succeeded in reproducing the general features of the folding pathway (see e.g. references [44], [45] for cytochrome c).

One conceptual refinement to arise from theoretical and simulation studies is the study of “good” reaction coordinates that correlate with commitment probability to complete the protein folding reaction [36], [46]–[51]. Reaction coordinates must generally take into account the energy surface on which the molecule of interest is undergoing conformational diffusion [52]–[54], and the Markovian or non-Markovian nature of the diffusion [55], [56]. In a system with many degrees of freedom on a complex energy landscape and obeying nontrivial steric restrictions, finding a best reaction coordinate or even a good reaction coordinate is a difficult task. Finding reaction paths between metastable minima is an old problem, in which many approaches have been developed to account for the underlying complex, multi-dimensional potential energy surface [57]–[63].

An alternate approach, in the spirit of defining order parameters in statistical and condensed matter physics, is to consider the geometry of the product and reactant in defining a reaction coordinate without reference to the underlying potential energy landscape. The overlap function 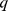 of a spin-glass is an example of a geometrically-defined order parameter [64], for which the underlying Hamiltonian determines behavior such as the temperature-dependence. We pursue such a geometric approach in this paper.

A transformation connecting unfolded states with the native folded state can be considered as a reaction coordinate. A transformation can also be used as a starting point for refinement, by examining commitment probability or other reaction coordinate formalism.

Several methods have been developed to find transformations between protein conformational pairs without specific reference to a molecular mechanical force field. These include coarse-grained elastic network models [65], [66], coarse-grained plastic network models [67], iterative cluster-normal mode analysis [68], restrained interpolation (the Morph server) [69], the FRODA method [70], and geometrical targeting (the geometrical pathways (GP) server) [71].

In this paper we consider transformations between polymer conformation pairs that would not be viable by a conjugate-gradient type or direct minimization approach, in that dead-ends would inevitably be encountered. We focus specifically on how one might find geometrically optimal transformations that account for polymer non-crossing constraints, which would apply to knotted proteins for example.

By a geometrically optimal transformation, we mean a transformation in which every monomer in a polymer, as represented by the 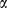-carbon backbone of a protein for example, would travel the least distance in 3-dimensional space in moving from conformation A to conformation B. This is a variational problem, and the equations of motion, along with the minimal transformation and the Euclidean distance covered, have been worked out previously [72]–[75]. Although minimal transformations have been found for the backbones of secondary structures, and the non-crossing problem has been treated [74], minimal transformations between unfolded and folded states for full protein chain lengths have not been treated before.

The minimal transformation inevitably involves curvilinear motion if bond, angle, or stereochemical constraints are involved [72], [76]. Such curvilinear transformations as a result of bond constraints were developed in [72]–[75]. If such constraints are neglected, the minimal distance corresponding to the minimal transformation reduces to the mean of the root squared distance (MRSD), or the mean of the straight line distances between pairs of atoms or monomers. This is not the conventional RMSD. For any typical pair of conformations, the MRSD is always less than the RMSD [73]. Used as an alignment cost function, aligned configurations using MRSD are globally different than those using RMSD [75]. The RMSD can be thought of as a least squares fit between the coordinates defining the two structures. Alternatively, it may also be thought of as the straight-line Euclidean distance between two structures in a high-dimensional space of dimension 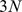, where 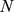 is the number of atoms in the protein, or 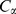 atoms if the protein is coarse-grained. Fast algorithms have been constructed to align structures using RMSD [77]–[82].

If several intermediate states are known along the pathway of a transformation between a pair of structures, then the RMSD may be calculated consecutively for each successive pair. This notion of RMSD as an order parameter goes back to reaction dynamics papers from the early 1980's [57]–[60], however in these approaches the potential energy governs the most likely reactive trajectories taken by the system, and RMSD is simply accumulated through the transition states.

In the absence of a potential surface except for that corresponding to steric constraints, the incremental RMSD may be treated as a cost function and the corresponding transformation between two structures found algorithmically [71]. However, the minimal transformation using RMSD (or 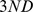 Euclidean distance) as a cost function is different than the minimal transformation using 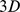 Euclidean distance (MRSD) as a cost function, and the RMSD-derived transformation does not correspond to the most straight-line trajectories. The RMSD is not equivalent to the total amount of motion a protein or polymer must undergo in transforming between structures, even in the absence of steric constraints enforcing deviations from straight-line motion. Conversely, the transformation corresponding to the MRSD will be curvilinear in the 3N-dimensional space.

In what follows, we develop a computational scheme for describing how difficult it might be for different proteins to reach their folded configuration. The essence is a calculation of how much “effort” the protein chain must expend to avoid having to cross through itself as it tries to realize its folded state. This involves finding the different ways a polymer can uncross or “untangle” itself, and then calculating the corresponding distance for each of the untangling transformations. Since there are typically several avoided crossings during a minimal folding transformation, finding the optimal untangling strategy corresponds to finding the optimal combination of uncrossing operations with minimal total distance cost.

After quantifying such a procedure, we apply this to full length protein backbone chains for several structural classes, including 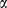-helical proteins, 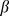-sheet proteins, 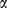-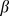 proteins, 2-state and 3-state folders, and knotted proteins. We generate unfolded ensembles for each of the proteins investigated, and calculate minimal distance transformations for each member of the unfolded ensemble to fold. From this calculation, we obtain the mean minimal distance to fold from the unfolded ensemble, for a given structural class. We look for differences in the mean minimal distance between structural and kinetic classes, and compare these to differences in other order parameters between the respective classes. The extra non-crossing distance per residue 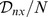 turns out to be the most consistent discriminator between different structural and kinetic classes of proteins. We find the extra distance covered to avoid chain crossing is generally a small fraction (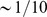) of the total motion. We also investigate how the various order parameters either correlate or are independent from each other.

We then select three proteins, an 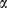-helical, a 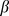-sheet, and a knotted protein, to further dissect the taxonomy of their minimal folding transformations. We construct what might be called “multiple transformation alignments” that describe the various different ways each protein can fold from an ensemble of unfolded conformations. We find that noncrossing motions of an N- or C-terminal leg are generally obligatory for a knotted protein, and only incidental for an 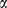 protein. A consensus minimal folding transformation is constructed for each of the above-mentioned native folds, and rendered schematically. By investigating a “pathway overlap” order parameter, we find that non-crossing constraints, as are prevalent in 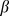 proteins and pervasive in knotted proteins, explicitly induce a pathway “mechanism” in protein folding, as defined by a common sequence of events independent of the initial unfolded conformation. We finally discuss our results and conclude.

## Methods

### Calculation of the transformation distance

The value of the uncrossing or non-crossing distance, 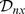, is calculated as follows: The chain transforms from conformation A to conformation B as a ghost chain, so the chain is allowed to pass through itself. The beads of the chain follow straight trajectories from initial to final positions. This is an approximation to the actual Euclidean distance 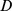 of the transformation, where straight line transformations of the beads are generally preceded or proceeded by non-extensive local rotations to preserve the link length connecting the beads as a rigid constraint [72], [73]. The instances of self-crossing along with their times are recorded. The associated cost for these crossings is computed retroactively, for example the distance cost for one arm of the chain to circumnavigate another obstructing part is then added to the “ghost” distance to compute the total distance.

The method for calculating the non-crossing distance 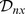 has three major components, evolution of the chain, crossing detection, and crossing cost calculation. Each are described in the subsections below.

#### Evolution of the chain

As mentioned above, the condition of constant link length between residues along the chain is relaxed, so that the non-extensive rotations that would generally contribute to the distance traveled are neglected here. This approximation becomes progressively more accurate for longer chains. Thus ideal transformations only involves pure straight-line motion. The approximate transformation is carried out in a way to minimize deviations from the true transformation (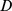), such that link lengths are kept as constant as possible, given that all beads must follow straight-line motion. We thus only allow deviations from constant link length when rotations would be necessary to preserve it; this only occurs for a small fraction of the total trajectory, typically either at the beginning or the end of the transformation [72], [73].

#### *A specific example*

**Figure 1:**
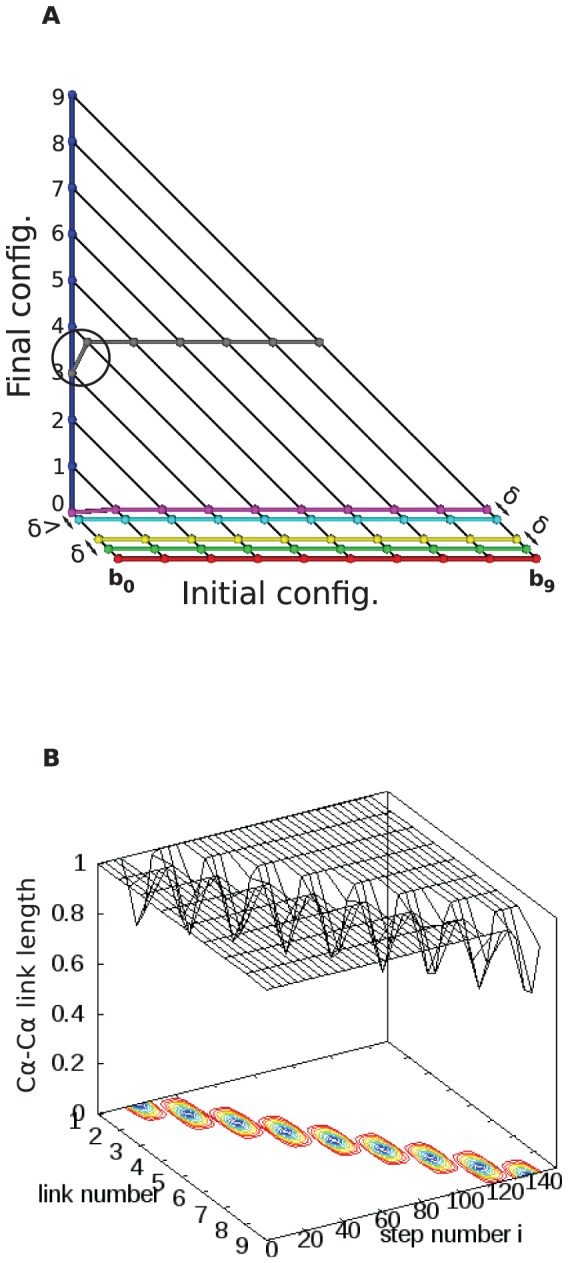
Approximate minimal transformation for a simple conformation pair, and the degree to which link length changes. (a) Several intermediate conformations for a transformation (A–G proceeding along the color sequence red, green, yellow, cyan, magenta, gray, and blue) are shown. The step-size delta is shown. Note the step in which the first bead of the chain (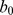) is “snapped” into the final conformation because its distance to the destination is less than 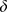 (going from D to E). In the intermediate conformation F (Gray), beads 0 to 3 have reached their final locations and no longer move. Note also the link length violation of link 4 in conformation F, due to the approximation that ignores end point rotations, for this intermediate figure. A milder violation is observed when going from D (cyan) to E (magenta), since bead 1 through 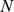 all assume a step size of 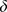 while bead 0 moved a step size 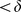. (b) Panel b shows a surface plot showing link length as a function of link number and step number during transformation. For the whole process, mean link length 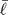 is 0.98 units and standard deviation 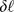 is 0.063.

As an example of the amount of distance neglected by this approximation, consider the pair of configurations in Figure 1, where a chain of 10 residues that is initially horizontal transforms to a vertical orientation as shown in the figure. The distance neglecting rotations (our approximation) is 77.78, in reduced units of the link length, while the exact calculation including rotations [72], [73] gives a distance of 78.56.

A few intermediate conformations are shown in the figure. In particular note the link length change (and hence violation of constant link length condition) in the fourth link for the gray conformation (conformation F), resulting from our approximation. If the link length is preserved, the transformation consists of local rotations at the boundary points.

Also note that when transforming from cyan to magenta the first bead moves less than 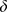, because it reaches its final destination and “sticks” to the final point, and will not be moved subsequently. A movie of this transformation is provided as Movie S1 in the Supporting Information.

#### General method

The algorithm to evolve the chain is as follows. Straight-line paths from the positions of the beads in the initial chain configuration to the corresponding positions of the beads in the final configuration are constructed. The bead furthest away from the destination, i.e. the bead whose path is the longest line, is chosen. Let this bead be denoted by index 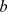 where 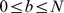. In the context of Figure 1, this bead corresponds to bead number 9 (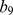). The bead is then moved toward its destination by a small pre-determined amount 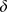, and the new position of bead 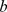 is recorded. In this way the transformation is divided into say 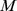 steps: 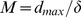, where 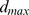 is the maximal distance. Let 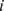 be the step index 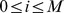. If initially the chain configuration was at step 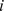 (e.g. 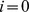), the spatial position of bead 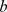 at step 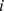 before the transformation 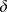 is denoted by 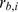, and after the transformation by 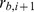. The upper bound 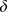 to capture the essence of the transformation dynamics differs according to the complexity of the problem. To capture all of the instances of self-crossing, a step size 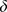 of two percent of the link length sufficed for all cases.

The neighboring beads (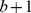 and 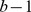) should also follow paths on their corresponding straight-line trajectories. Their new position on their paths (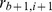 and 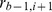) are then calculated based on the constant link length constraints. This new position corresponds to moving the beads by 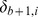, 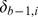 respectively. Once 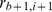 and 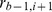 are calculated, we proceed and calculate 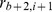 and 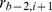 until we reach the end points of the chain. As an example consider Figure 1, going from the conformation B (Green) to the conformation C (Yellow). First, bead number 9, which is the bead farthest away from its final destination, is moved by 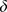, then taking constant link length constraints and straight line trajectories into account, the new position of bead 8 is calculated and so on, until all the new bead positions which correspond to the yellow conformation are calculated.

If somewhere during the propagation to the endpoints, a solution cannot be constructed or no continuous solution exists, i.e. 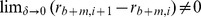, then we set 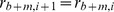. That is, the bead will remain stationary for a period of time [83]. Consequently 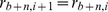 for all beads with 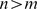 that have not yet reached their final destination. This is because the new position of each bead is calculated by the position of the bead next to it for any particular step 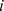. The same recipe is applied when propagating incremental motions 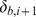 along the other direction of the chain (going from 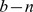 to 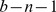) as well. When a given bead that has been held stationary becomes the furthest bead away from its final position, it is then moved again. I.e. stationary beads can move again at a later time during the transformation if they become the furthest beads away from the final conformational state. Such a scenario does not occur in the context of the simple example of Figure 1, however in Movie S2 in the Supporting Information, a transformation is given for a full protein that involves such a process. During the course of such a transformation the viewer will notice that several beads on the chain (in the upper right in the movie) remain stationary for a part of the transformation. For these beads no continuous solution for the motion exists, i.e. as 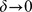 the beads in question cannot move without violating the constant link length constraint. At a later time during the transformation, when the beads in the given segment are farthest from the final folded conformation, the beads resume motion.

Once the positions of all the beads in step 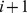 are calculated, the same procedure is repeated for step 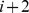 and so on, until the chain reaches the final configuration. If the position of a given bead 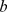 at step 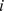 is such that 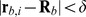, where 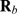 is the spatial position of bead 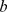 in the final conformation, then 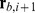 is set to 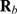. In other words we discretely snap the bead to the final position if it is closer than the step size 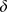. In the context of Figure 1, this corresponds to going from conformation D (Cyan) to conformation E (Magenta). Bead 0 (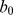) is snapped to the final conformation. Once a bead reaches its destination it locks there and will never move again. See conformation F (gray) in Figure 1.

**Figure 2:**
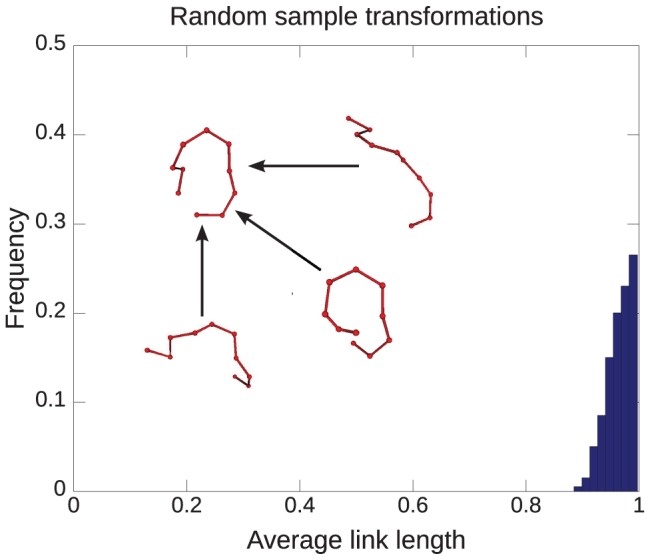
Link length statistics for randomly generated transformation pairs. Histogram of the average link length over the course of a transformation, for transformations between 200 randomly generated structures of 9 links and the (randomly generated) reference structure shown in the inset to the figure. The “native” or reference state is shown in the inset, along with several of the 200 initial states. For the ensemble of transformations shown, the ensemble average of the mean link length is 0.96.

Figure 2 shows a histogram of the mean link length over the course of a transformation, for 200 transformations between random initial structures generated by self avoiding random walks (SAW), and one pre-specified SAW. The length of the random chains was 9 links. The chains were aligned by minimizing MRSD before the transformation took place [73]–[75], where MRSD stands for the mean root squared distance and is defined by 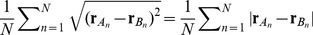. Deviations from the full unperturbed link length are modest: the ensemble-averaged mean link length is 96% of the initial link length.

#### Crossing Detection

**Figure 3:**
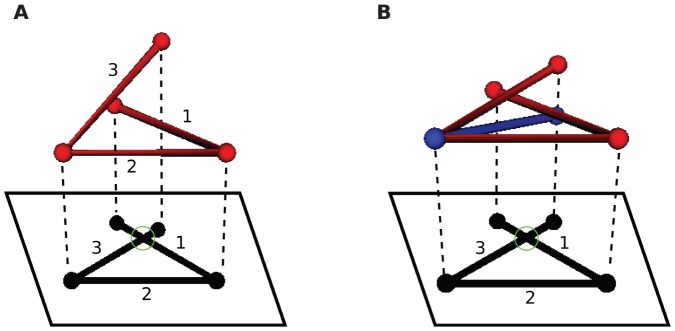
Crossing detection using projections. (a) A 3 link chain with its vertical projection. A crossing in the projection is shown with a green circle. The crossing in the projection occurs at points 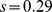 and 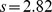, where the chain is parametrized uniformly from 0 to 3. Since link 1 is under link 3 at the point of projection crossing, 0.29 will appear with a negative sign in the corresponding 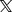 (eqn 1). (b) The blue chain and the red chain have the exact same vertical projection, however their corresponding 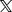 matrices are different in sign, as given in Eq. 2. This indicates that the over-under sense has changed for the links whose projections are crossing. This in turn indicates that a true crossing has occurred when going from the red conformation to the blue conformation, as opposed to a series of conformations where the chain has navigated to conformations having the opposite crossing sense without passing through itself.

As stated earlier, during the transformation the chain is initially treated as a ghost chain, and so is allowed to cross itself. To keep track of the crossing instances of the chain, a crossing matrix 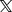 is updated at all time steps during the transformation. If the chain has 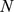 beads and 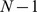 links, we can define an 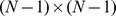 matrix 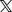 that contains the crossing properties of a 2D projection of the strand, in analogy with topological analysis of knots. The element 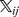 is nonzero if link 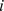 is crossing link 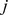 in the 2D projection at that instant. Without loss of generality we can assume that the projection is onto the XY plane, as in Figure 3. We illustrate the independence of our method on projection plane explicitly for a crossing event in cold-shock protein (1CSP) in File S1 (see figure SA). We use the XY plane projection throughout this paper [84].

We parametrize the chain uniformly and continuously in the direction of ascending link number by a parameter 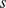 with range 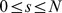. So for example the middle of the second link is specified by 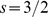. If the projection of link 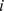 is crossing the projection of link 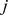, then 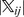 is the value of 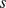 at the crossing point of link 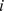 and 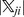 is the value of 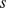 at the crossing point of link 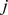. If link 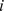 is over 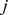 (i.e. the corresponding point of the cross on link 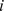 has a higher 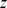 value than the corresponding point of the cross on link 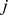) then 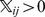, otherwise 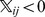. Thus after the sign operation, 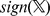 is an anti-symmetric matrix.

A simple illustrative example of the value of 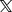 for the 3-link chain in Figures 3a and 3b is
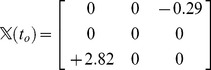
(1a)

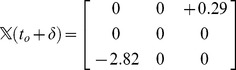
(1b)The fact that 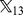 is negative at time 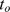 indicates that at that instant, link 1 is under link 3 in 3D space, above the corresponding point on the plane on which the projections of the links have crossed (green circle in Figure 3).

At each step during the transformation of the chain, the matrix 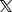 is updated. A true crossing event is detected by looking at 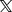 for two consecutive conformations. A crossing event occurs when any non-zero element in the matrix 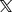 discontinuously changes sign without passing through zero. Once 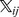 changes sign, 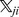 must change sign as well. If the chain navigates through a series of conformations that changes the crossing sense and thus the sign of 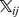, but does not pass through itself in the process, the matrix elements 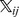 will not change sign discontinuously but will have values of zero at intermediate times before changing sign.

Movie S3 in the Supporting Information shows the result of applying crossing detection. In the movie of the transformation, whenever an instance of self-crossing is detected, the transformation is halted and the image is rotated to make the location of the crossings easier to visualize.

#### Crossing Cost calculation

Even in the simplest case of crossing, there are multiple ways for the real chain to have avoided crossing itself. The extra distance that the chain must have traveled during the transformation to respect the fact that the chain cannot pass through itself is called the “non-crossing” distance 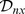. If the chain were a ghost chain which could pass through itself, the corresponding distance for the whole transformation would be the MRSD, along with relatively small modifications that account for the presence of a conserved link length. Accounting for non-crossing always introduces extra distance to be traveled.

**Figure 4:**
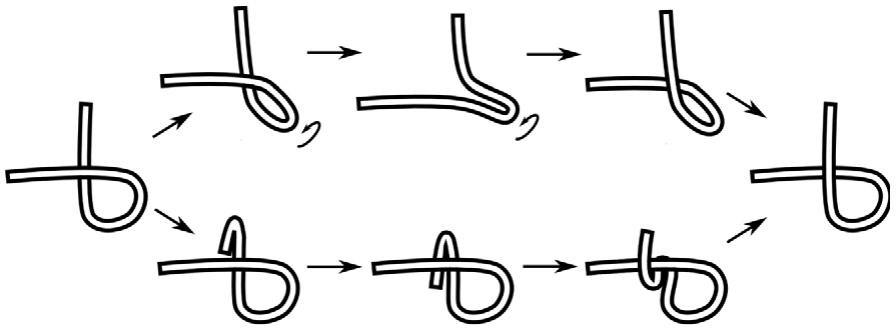
Two possible minimal uncrossing transformations. Two possible untangling transformations. The top transformation involves twisting of the loop. The lower transformation involves a snake like movement of the vertical leg. A third one would involve moving the horizontal leg, in a similar snake-like fashion. Note that the moves represented here are not necessarily the most efficient ones in their topological class, but rather the most intuitive ones. There are transformations that are topologically equivalent but generally involve less total motion of the chain (see for example Figures 11(a), 11(b)).

As the chain is transforming from conformation A to conformation B as a ghost chain according to the procedure discussed above, a number of self-crossing incidents occur. Figure 4 shows a continuous but topologically equivalent version of the crossing event shown in Figure 3 (b). Even for this simple case, there are multiple ways for the transformation to have avoided the crossing event, each with a different cost.

**Figure 5:**
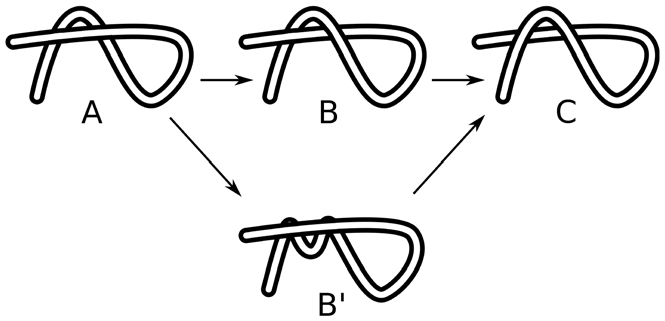
Accounting for history-dependence in minimal uncrossing transformations. The minimal untangling movement in going from A to C (through B′) is less than the sum of the minimum untangling movements going from A to B and then from B to C.

Furthermore, later crossings can determine the best course of action for the previous crossings. Figure 5 illustrates how non-crossing distances are non-additive, so that one must look at the whole collection of crossing events. Therefore to find the optimum way to “untangle” the chain (reverse the sense of the crossings), one must look at all possible uncrossing transformations, in retrospect. The recipe we follow is to evolve the chain as a ghost chain and write down all the incidents of self-crossings that happen during the transformation. Then looking at the global transformation, we find the best untangling movement that the chain could have taken.

To compute the extra cost introduced by non-crossing constraints we proceed as follows: We construct a matrix that we call the cumulative crossing matrix 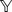. 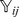 is non-zero if link 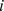 has truly (in 3D) crossed link 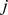, at any time during the transformation. This matrix is thus conceptually different than the matrix 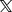, which holds only for one instant (one conformation) and which can have crossings in the 2D projection which are not true crossings during the transformation. The values of the elements of 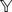 are calculated in the same way that the values are calculated for 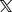. The sign also depends on whether the link was crossed from over to under or from under to over, so that a given projection plane is still assumed. The order in which the crossing have happened are kept track of in another matrix 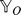. The coordinates of all the beads at the instant of a given crossing are also recorded. For example, if during the transformation of a chain, two crossing have happened, then two sets of coordinates for intermediate states are also stored. We describe a simple concrete example to illustrate the general method next.

#### *A Concrete Example*

**Figure 6:**
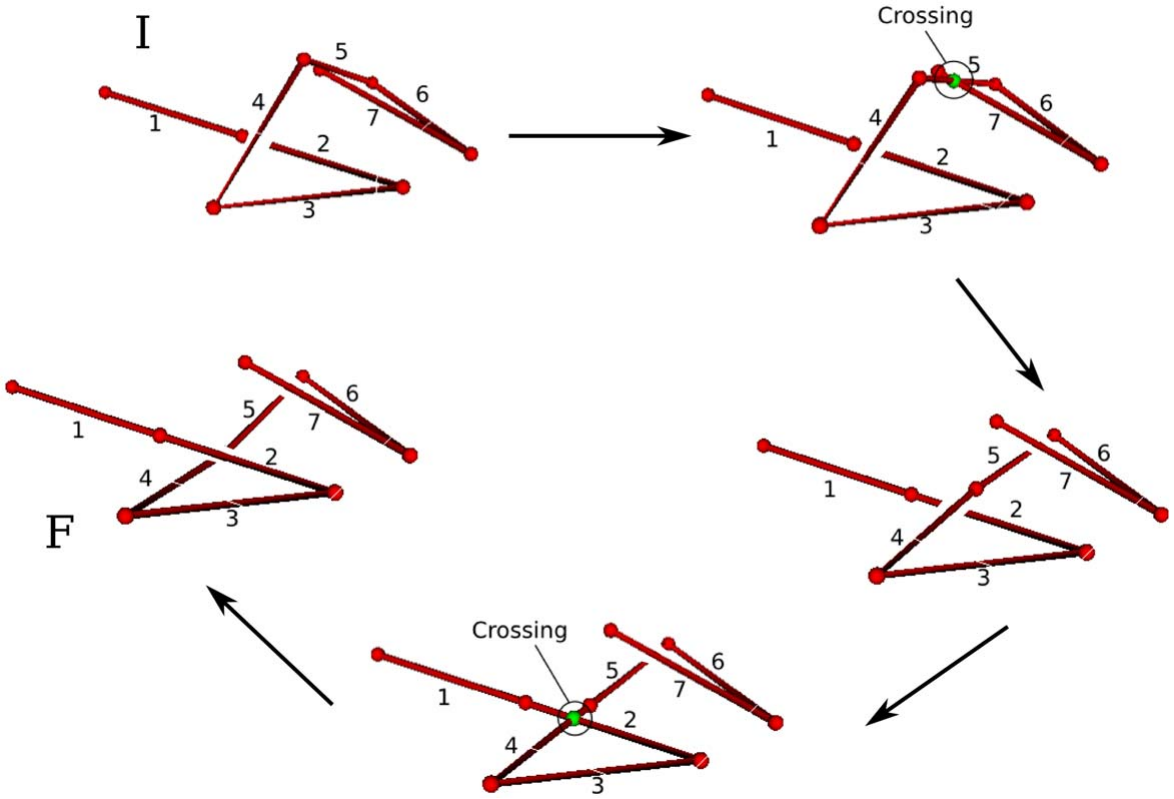
Snapshots of a transformation with two crossings. A few snapshots during a transformation involving 2 instances of chain crossing. The transformation occurs clockwise starting from initial configuration I and proceeding to final configuration F.

Figure 6 shows a simple transformation of a 7-link chain. During the transformation the chain crosses itself in two instances. The first instance of self-crossing is between link 5 and link 7. The second instance is when link 2 crosses link 4. The location of the crossing along the chain is also recorded: i.e. if we assume that the chain is parametrized by 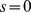 to 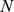, then at the instant of the first crossing (link 5 and link 7) 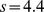 (link 5) and 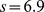 (link 7). The second crossing occurs at 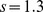 (link 2) and 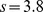 (link 4). The full coordinates of all beads are also known: we separately record the full coordinates of all beads at each instant of crossing. The information that indicates which links have crossed and their over-under structure can be aggregated into the cumulative crossing matrix 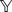. For the example in Figure 6, the cumulative matrix (up to a minus sign indicating what plane the crossing events have been projected on) is
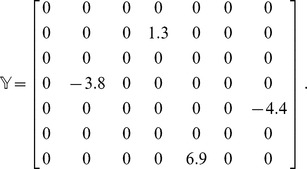


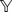
 tells us, during the whole process of transformation, which links have truly crossed one another and what the relative over-under structure has been at the time of crossing. For example, by glancing at the matrix we can see that two links 5,7 and 2,4 have crossed one another. We also know from the sign of the elements in 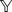 that both links 2 and 7 were underneath links 4 and 5 just prior to their respective crossings in the reference frame of the projection. Two links will cross each other at most once during a transformation. If one link, e.g. link 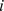, crosses several others during the transformation, elements 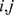, 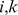 etc… along with their transposes will be nonzero.

The order of crossings can be represented in a similar fashion as a sparse matrix.
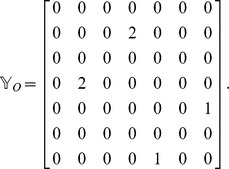
Analyzing the structure of the crossings is similar to analyzing the structure of a knot, wherein one studies a knot's 2D projections, noting the crossings and their over/under nature based on a given directional parameterization of the curve [89]–[91]. One difference here is that we are not dealing with true closed-curve knots (in the mathematical sense), as a knot is a representation of S1 in S3. Here we treat open curves.

#### Crossing substructures

By studying the crossing structure of open-ended pseudo-knots in the most general sense, one can identify a number of sub-structures that recur in crossing transformations. Any act of reversing the nature of all the crossings of the polymer can be cast within the framework of some ordered combination of reversing the crossings of these substructures.

We identify three sub-structures: Leg, Loop, and Elbow.

#### *Leg*

**Figure 7:**
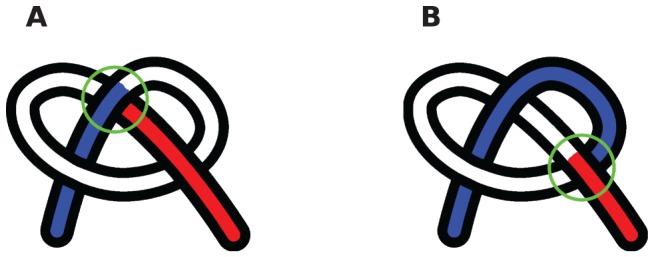
Identification of leg-uncrossing. For the crossing points indicated by the green circles, two legs, colored blue and red, can be identified. Each leg starts at the crossing and terminates at an end.

**Figure 8:**
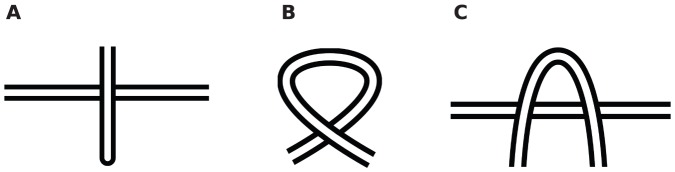
Crossing substructures. (a) A single leg structure, (b) A loop structure, (c) An elbow structure.

Given any self-crossing point of a chain, a leg is defined from that crossing point to the end of the chain. Therefore for each self-crossing point two legs can identified as the shortest distance along the chain from that crossing point to each end—see Figure 7. A single leg structure is shown in Figure 8(a).

#### *Loop*

As stated earlier, when traveling along the polymer one arrives at each crossing twice. If the two instances of a single crossing are encountered consecutively while traveling along the polymer, and no intermediate crossing occurs, then the substructure that was traced in between is a loop. See Fig. 8(b).

#### *Elbow*

If two consecutive crossings have same over-under sense, then they form an elbow; see Fig. 8(c). Note that the same two consecutive crossing instances will occur in reverse order on the second visit of the crossings: these form a dual of the elbow. By convention the segment with longer arc-length between the two consecutive crossings is defined as the elbow. This would be the horseshoe shaped strand in Figure 8(c).

#### Reversing the crossing nature

The goal of this formalism is to assist in finding a series of movements that will result in reversing the over-under nature of all the crossings, with the least amount of movement required by the polymer. So at this point we introduce basic movements that that will reverse the nature of the crossings for the above substructures.

#### *Using leg movement*

**Figure 9:**
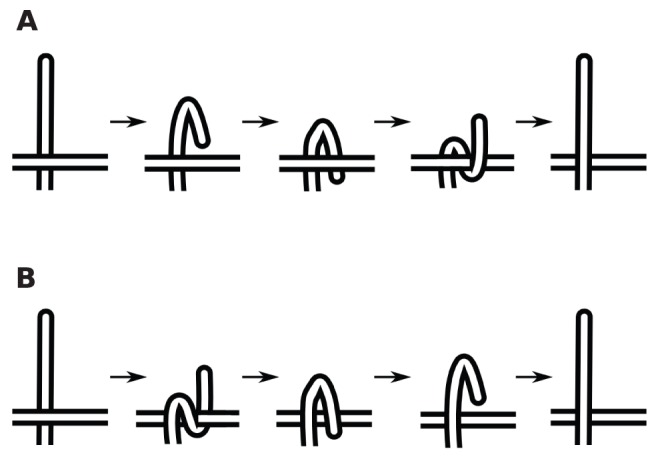
Schematic illustration of the canonical leg movement. Schematic illustration of the canonical leg movement, either from left to right as in (a) or effectively its time reverse as in (b). Both transformations traverse the same distance. The transformation in (a) is equivalent to the “plug” transformation analyzed in the context of folding simulations for trefoil knotted proteins [134], while the transformation in (b) (see ref. [74] for a detailed description of this transformation) is equivalent to the “slipknotting” transformation more often observed in the folding of knotted proteins [138].

A transformation that reverses the over/under nature of a leg involves the motion of all the beads constituting the leg. Each bead must move to the location of the crossing (the “root” of the leg), and then move back to its original location [74]. The canonical leg movement is shown schematically in Figure 9.

**Figure 10:**
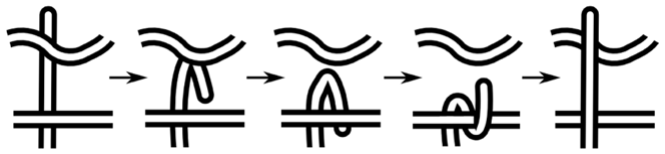
A single leg movement can undo several crossings. One can reverse the over-under nature of all the crossings that have occurred on a leg, through a single leg movement.

We can reverse the nature of all the crossings that have occurred on a leg, if more than one crossing occurs, through a leg movement (see Figure 10). The move is topologically equivalent to the movement of the free end of the leg along the leg up to the desired crossing, and then moving all the way back to the original position while reversing the nature of the crossing on the way back.

#### *Loop twist and loop collapse*

**Figure 11:**
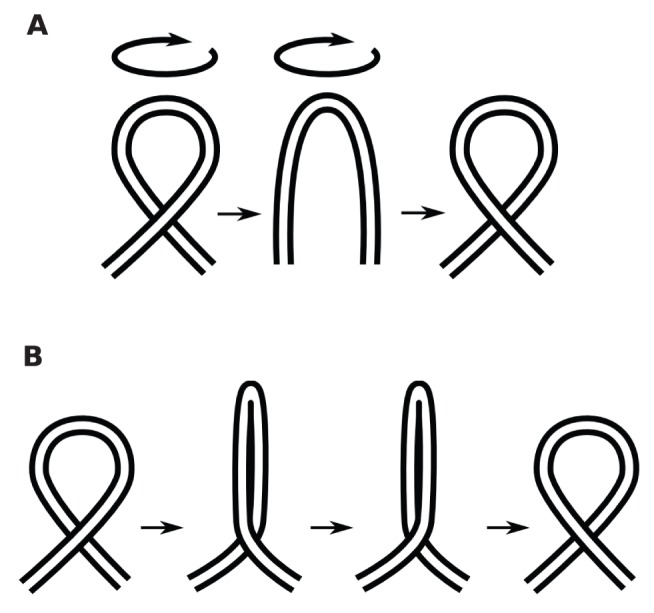
Relation of minimal loop uncrossing to Reidemeister type I moves. (a)Reversing the over-under nature of a crossing through a topological loop twist: Reidemeister move type I. (b) By “pinching” the loop before the twist, the cost in distance for changing the crossing nature is reduced.

Reversing the crossing of a loop substructure can be achieved by a move that is topologically equivalent to a twist, see Figure 11 (a). This type of move is called a Reidemeister type I move in knot theory. However the optimal motion is generally not a twist or rotation in 3-dimensional space (3D). Figure 11(b) shows a move which is topologically equivalent to a twist in 3D, but costs a smaller distance, by simply moving the residues inside the loop in straight lines to their final positions, resulting in a “pinching” motion to close the loop and re-open it. From now on we refer to the optimal motion simply as loop twist, because it is topologically equivalent, but we keep in mind that the actual optimal physical move, and the distance calculated from it, is different.

#### *Elbow moves*

**Figure 12:**
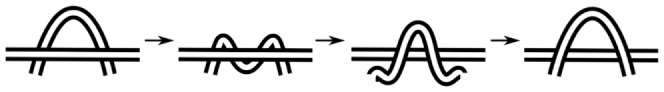
Schematic of the canonical elbow move. Schematic of the canonical elbow move. From left to right.

Reversing the crossings of an elbow substructure can be done by moving the elbow segment in the motion depicted in Figure 12: Each segment moves in a straight line to its corresponding closest point on the obstruction chain, and then it moves in a straight line to its final position.

#### Operator Notation

The transformations for leg movement, elbow, and twist can be expressed very naturally in terms of operator notation, where in order to untangle the chain the various operators are applied on the chain until the nature of all the self-crossing are reversed.

If we uniquely identify each instance of self-crossing by a number, then a topological loop twist at crossing 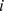 can be represented by the operator 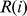 (*R* for Reidemeister). An elbow move, for the elbow defined by crossings 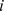 and 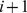, can be represented as 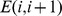. As discussed above, for each self-crossing, two legs can be identified corresponding to the two termini of the chain. This was exemplified in Figure 7, by the red and blue legs. Since we choose a direction of parametrization for the chain, we refer to the two leg movements as the “start leg” movement and the “end leg” movement, and for a generic crossing 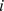 we denote them as 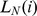 and 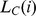 respectively.

The operators that we defined above are left acting (similar to matrix multiplication). So a loop twist at crossing 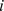 followed by an elbow move at crossings 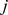 and 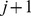 is represented by 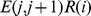.

#### *Example*

**Figure 13:**
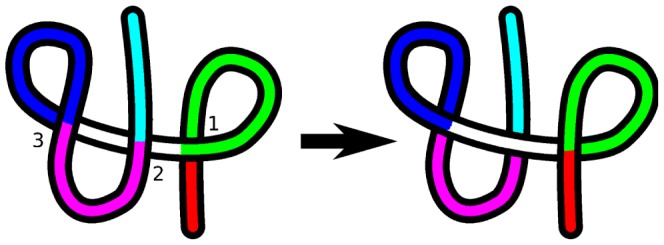
A simple example depicting various crossing substructures. A chain with several self-crossing points before and after untangling. Various topological substructures that are discussed in the text are color coded. For the case of the legs (red and cyan) note that various other legs can be identified, for example a leg that starts at crossing 2 and ends at the red terminus. Here we color only the shortest legs from crossing 1 to the terminus as red, and crossing 2 to the opposite terminus as cyan.

Figure 13 shows sample configurations before and after untangling. The direction of parametrization is from the red terminus to the cyan terminus. It can be seen that there are several ways to untangle the chain. One example would be 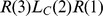, which consists of a twist of the green loop, followed by the cyan leg movement, followed by a twist of the blue loop. Another path of untangling would be 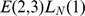, which is movement of the red leg followed by the magenta elbow move.

For the two above transformations, the order of operations can be swapped, i.e. they are commutative, and the resulting distance for each of the transformations will be the same. That is 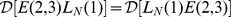. However, 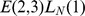 is a more efficient transformation than 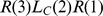, i.e. 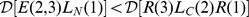.

Other transformation moves are not commutative in the algorithm, for example in Figure 13, 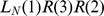 is not allowed, since 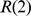 will only act on loops defined by two instances of a crossing that are encountered consecutively in traversing the polymer, i.e. no intermediate crossings can occur. Therefore even if crossing 2 happens kinetically before crossing 3 during the ghost transformation, only transformation 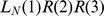 is allowed in the algorithm.

#### Minimal uncrossing cost

**Figure 14:**
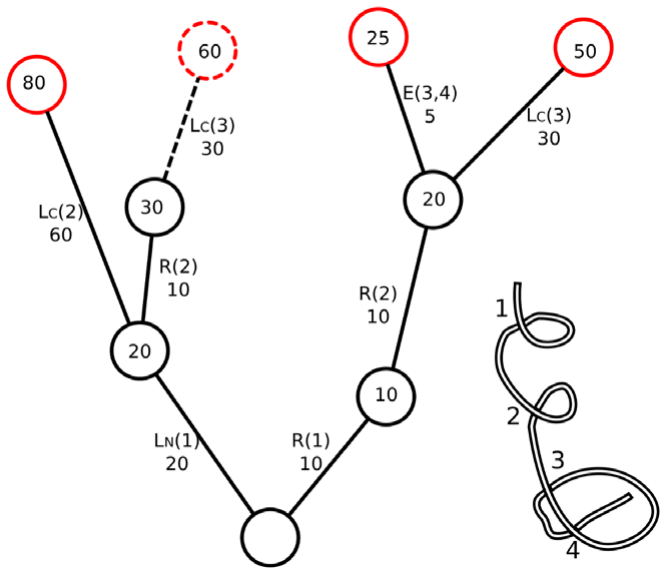
Illustration of the depth-first tree search algorithm for the given crossing structure shown. An example (subset) tree of possible transformations for a given crossing structure. Accumulated distances are given inside the circles representing nodes of the tree; the non-crossing transformations and their corresponding distances are shown next to the branches of the tree. The algorithm starts from the bottom node and proceeds to the top nodes, starting in this case along the right-most branch. The possible transformations to be considered as candidate minimal transformations are : 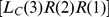, 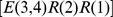, 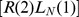 which then terminates because the accumulated distance exceeds the minimum so far of 25, and 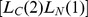.

For each operator in the above formalism, a transformation distance/cost can be calculated. Hence the optimal untangling strategy is finding the optimal set of operator applications with minimal total cost. This solution amounts to a search in the tree of all possible transformations, as illustrated in Figure 14. The optimal application of operators can be computed by applying a version of the depth-first tree search algorithm.

According to the algorithm, from any given conformation there are several moves that can be performed, each having a cost associated with the move. The pseudo-code for the search algorithm can be written as follows:
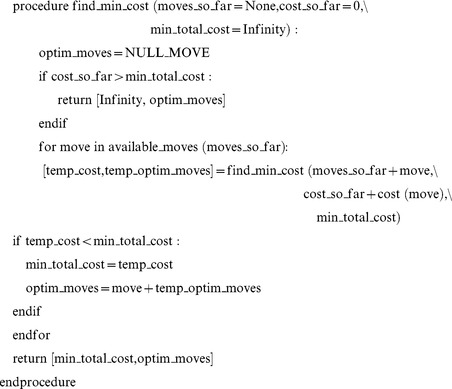
The values to the right side of the equality sign in the arguments of the procedure are the default values that the procedure starts with. The procedure is called recursively, and returns both the set of optimal uncrossing moves (for a given crossing matrix corresponding to a starting and final conformation), and the distance corresponding to that set of optimal uncrossing moves.

The algorithm visits all branches of the tree of possible uncrossing operations until it reaches the end. However it is smart enough to terminate the search along the branch if the cost of operations exceeds that of a solution already found. See Figure 14 for an illustration of the depth-first search tree algorithm. The above procedure was implemented using both the GNU Octave programming language and C++. To optimize speed by eliminating redundant moves, only one permutation was considered when operators commuted.

### Generating unfolded ensembles

To generate transformations between unfolded and folded conformations, we adopt an off-lattice coarse grained 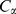 model [92], [93], and generated an unfolded structural ensemble from the native structure as follows. For a native structure with 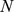 links, we define three data sets:

- The set of 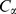 residue indices 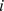, for which 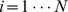

- The set of native link angles 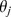 between three consecutive 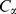 atoms, for which 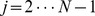

- The set of native dihedral angles 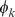 between four consecutive 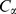 atoms, i.e. the angle between the planes defined 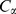 atoms (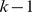, 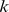, and 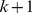), and (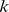, 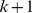, and 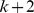). The index 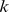 runs from 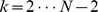.

The distribution of C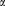-C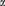 distances in PDB structures is sharply peaked around 3.76 Å (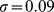
Å). In practice we took the first C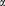-C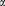 distance from the N-terminus as representative, and used that number for the equilibrium link length for all C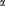-C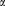 distances in the protein.

To generate an unfolded ensemble, we start by selecting at random a 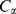 atom 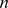 (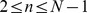) in the native conformation, and we then perform rotations that change the angle centered at that randomly chosen residue 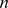, 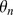, and that change the dihedral defined by rotations about the bond 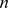-(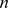
+1), 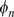. If 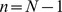 only the angle is changed. The new angle and dihedral are selected at random from the Boltzmann distributions as described below. After each rotation, 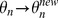 and 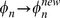. Changing these angles rotates the entire rest of the chain, i.e. all the beads 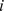 with 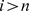 are rotated to a new position. This recipe corresponds to an extension of the pivot algorithm [94], [95].

However, we additionally require that the values of each angle and dihedral that are present in the native structure, 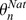 and 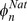, are more likely to be observed. We implement this criterion in the following way. The new angle 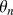 is chosen from a probability distribution proportional to 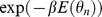, where 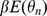 is computed from:
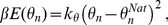
(2)where we have set 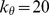. Similarly for the dihedral 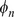, the probability distribution function is proportional to 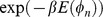, where 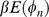 is computed from
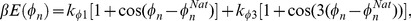
(3)where 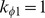, and 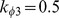. The fact that the 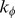
s are much smaller than 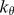 means that for a given temperature, dihedral angles are more uniformly distributed than bond angles. If all 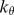 and 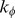 are set to zero, then all states are equally accessible and the algorithm reduces to the pivot algorithm, i.e. a generator for unbiased, self-avoiding random walks. If all 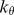 and 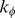 are set to 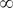, then chain behaves as a rigid object and does not deviate from its native state.

Each pivot operation results in a new structure that must be checked so that it has no steric overlap with itself, i.e. the chain must be self-avoiding. If the new chain conformation has steric overlap, then the attempted move is discarded, and a new residue is selected at random for a pivot operation.

In practice, we defined steric overlap by first finding an approximate contact or cut-off distance for the coarse-grained model. The contact distance was taken to be the smaller of either the minimum 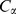-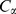 distance between those residues in native contact (where two residues are defined to be in native contact if any of their heavy atoms are within 4.9 Å), or the 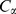-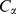 distance between the first two consecutive residues. For SH3 for example the minimum 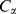 distance in native contacts is 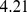
Å and the first link length is 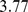
Å, so for SH3 all non-neighbor beads must be further than 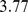
Å for a pivot move to be accepted. Future refinements of the acceptance criteria can involve the use of either the mean C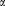-C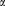 distance or other criteria more accurately representing the steric excluded volume of residue side chains.

In our recipe, to generate a single unfolded structure we start with the native structure and implement 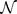 *successful* pivot moves, where 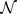 is related to the number of residues 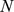 by 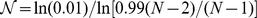.

For the next unfolded structure we start again from the native structure and pivot 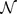 successful times, following the above recipe. Note that 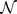 successful pivots does not generally affect all beads of the chain. In the most likely scenario some beads are chosen several times and some beads are not chosen at all, according to a Poisson distribution. This particular choice of 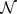 means that for polymers with 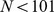 where 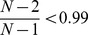, the chance that any given link is not pivoted at all during the 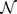 pivot operations is 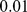. On the other hand for longer polymers where 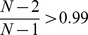, the probability that any particular segment of the protein with the length 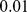 of the total length, has 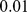 chance of not having any of its beads pivoted. For any 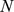 however, the shear number of pivot moves generally ensures a large RMSD between the native and generated unfolded structures.

Each unfolded structure generally retains small amounts of native-like secondary and tertiary structure, due to the native biases in angle and dihedral distributions. For example, for SH3 the number of successful pivot moves was 162 and the mean fraction of native contacts in the generated unfolded ensemble was 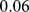.

### Protein dataset

The 45 proteins used in this study are given in Table 1. When divided into kinetic classes, they consist of 25 2-state folders, 13 non-knotted 3-state folders, and 7 knotted proteins not used in the kinetic analysis. Structurally there are 11 all 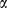-helix proteins, 14 all 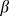-sheet proteins, 13 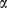-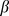 proteins, and 5 knotted proteins. These proteins were selected randomly from the datasets in references [96], [97], where kinetic rate data was available to categorize the proteins into 2-state or 3-state folders. Our dataset contains 27 out of the 52 proteins in [96], and 38 out of the 72 proteins in [97]. The datasets in [96], [97] do not include knotted proteins however; the Knotted proteins were taken from several additional sources, including references [98] (1NS5) [99], (1MXI) [100], (3MLG) [101], (2K0A) [102], (2EFV), and the protein knot server KNOTS [103] (1O6D, 2HA8). Aside from the Stevadore knot in [102] we did not consider pseudo-knots more complex than the 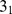 trefoil.

**Table 1:** Proteins studied in this paper.

PDB	x-State	2ndry str.	LRO	RCO	ACO	MRSD	RMSD	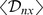	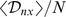	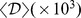	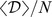	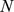
1A6N	3	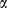 -helix	1.4	0.1	14.0	26.2	29.2	285	1.9	4.24	28.1	151
1APS	2	Mixed	4.2	0.2	21.8	22.7	25.4	201	2.1	2.43	24.8	98
1BDD	2	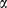 -helix	0.9	0.1	5.2	14.0	14.9	76.5	1.3	0.91	15.2	60
1BNI	3	Mixed	2.5	0.1	12.3	20.8	22.8	209	1.9	2.46	22.8	108
1CBI	3	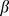 -sheet	2.8	0.1	18.8	25.1	27.9	286	2.1	3.70	27.2	136
1CEI	3	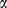 -helix	1.0	0.1	9.1	16.7	18.9	71.4	0.8	1.49	17.5	85
1CIS	2	Mixed	3.3	0.2	10.8	15.1	16.8	99.7	1.5	1.10	16.6	66
1CSP	2	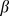 -sheet	3.0	0.2	11.0	16.8	18.4	98.0	1.5	1.23	18.3	67
1EAL	3	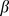 -sheet	2.5	0.1	15.7	24.9	27.9	278	2.2	3.44	27.1	127
1ENH	2	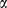 -helix	0.4	0.1	7.4	13.5	14.9	28.0	0.5	0.76	14.1	54
1G6P	2	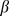 -sheet	3.8	0.2	11.7	16.4	18.0	83.1	1.3	1.17	17.7	66
1GXT	3	Mixed	3.7	0.2	18.6	21.1	23.5	148	1.7	2.03	22.8	89
1HRC	2	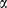 -helix	2.2	0.1	11.7	19.6	22.2	126	1.2	2.17	20.8	104
1IFC	3	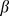 -sheet	2.8	0.1	17.7	25.1	27.9	284	2.2	3.58	27.3	131
1IMQ	2	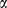 -helix	1.7	0.1	10.4	16.1	17.9	80.7	0.9	1.46	17.0	86
1LMB	2	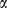 -helix	1.1	0.1	7.1	17.0	18.6	76.8	0.9	1.55	17.9	87
1MJC	2	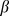 -sheet	3.0	0.2	11.0	17.5	19.2	110	1.6	1.32	19.1	69
1NYF	2	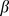 -sheet	2.8	0.2	10.6	15.3	17.0	87.4	1.5	0.97	16.8	58
1PBA	2	Mixed	2.6	0.1	12.0	18.9	20.8	156	1.9	1.69	20.8	81
1PGB	2	Mixed	2.1	0.2	9.7	14.1	15.7	25.4	0.5	0.81	14.5	56
1PKS	2	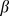 -sheet	3.8	0.2	15.2	17.9	20.2	136	1.8	1.50	19.7	76
1PSF	3	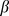 -sheet	2.8	0.2	11.7	16.8	19.4	72.1	1.0	1.23	17.8	69
1RA9	3	Mixed	3.4	0.1	22.3	25.5	28.6	402	2.5	4.46	28.1	159
1RIS	2	Mixed	3.0	0.2	18.4	21.5	23.9	163	1.7	2.25	23.2	97
1SHG	2	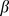 -sheet	3.0	0.2	10.9	15.1	16.7	92.3	1.6	0.95	16.7	57
1SRL	2	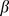 -sheet	3.1	0.2	11.0	14.8	16.3	94.5	1.7	0.92	16.5	56
1TIT	3	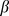 -sheet	4.1	0.2	15.8	18.7	20.8	154	1.7	1.82	20.4	89
1UBQ	2	Mixed	2.4	0.2	11.5	17.0	18.9	92.1	1.2	1.39	18.2	76
1VII	2	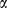 -helix	0.4	0.1	4.0	8.1	9.2	4.1	0.1	0.30	8.2	36
1WIT	2	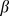 -sheet	5.0	0.2	18.9	20.4	22.7	168	1.8	2.07	22.2	93
2A5E	3	Mixed	2.6	0.1	8.3	22.2	23.9	354	2.3	3.82	24.5	156
2ABD	2	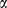 -helix	2.3	0.1	12.0	18.2	20.0	77.5	0.9	1.65	19.1	86
2AIT	2	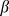 -sheet	4.1	0.2	14.4	16.9	18.7	107	1.5	1.36	18.3	74
2CI2	2	Mixed	2.7	0.2	10.0	15.1	16.9	78.3	1.2	1.06	16.4	65
2CRO	3	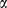 -helix	1.2	0.1	7.3	14.0	15.5	37.3	0.6	0.95	14.6	65
2HQI	2	Mixed	4.3	0.2	13.6	16.3	18.4	86.9	1.2	1.26	17.5	72
2PDD	2	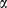 -helix	1.0	0.1	4.8	10.6	11.5	19.9	0.5	0.48	11.0	43
2RN2	3	Mixed	3.6	0.1	19.3	27.7	30.9	521	3.4	4.81	31.0	155
1O6D	–†	Knotted	3.1	0.1	18.9	26.2	28.7	515	3.5	4.36	29.7	147
2HA8	–†	Knotted	3.3	0.1	16.2	25.7	28.5	671	4.1	4.84	29.9	162
2K0A	–†	Knotted	3.4	0.1	14.6	22.4	24.5	369	3.4	2.81	25.8	109
2EFV	–†	Knotted	2.1	0.2	12.6	20.0	21.8	147	1.8	1.79	21.8	82
1NS5	3	Knotted	2.9	0.1	18.2	27.5	30.4	503	3.3	4.71	30.8	153
1MXI	3	Knotted	2.8	0.1	16.7	26.1	29.0	643	4.0	4.85	30.1	161
3MLG	3	Knotted	1.2	0.1	21.4	27.7	30.8	481	2.8	5.16	30.5	169

† Data not available at present.

Several of these proteins (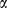-amylase inhibitor 2AIT and MerP mercuric ion binding protein 2HQI) have disulfide bonds present in the native structure. These constraints are not used in the current analysis. The folding pathways we obtain may be thought of as relevant to the initial folding event before disulfide bonds are formed, or for a protein of equivalent topology but sequence lacking the disulfide bond. Lack of preservation of disulfide bonds is a shortcoming of the present algorithm; development of more accurate computational algorithms for unfolded ensemble generation are a topic of future work.

Several of the proteins also have ligands present in the crystal or NMR structures. These include 1A6N and 1HRC (heme ligands), 1RA9 (Nicotinamide adenine), 1GXT (sulfate), 1MXI (iodide ion), 2K0A (3 Zn ions), 2EFV (phosphate ion). Since we have removed energetics in general from our analysis of geometrical pathways, these ligands and any effect they may have on the folding pathway due to protein-ligand interactions are not included here. In the folding kinetics analysis of references [96], [97], they are generally not present either, e.g. the folding rate for 1A6N is actually that for apomyoglobin [104].

#### Structural alignment properties of our protein dataset

To categorize proteins as two- or three-state, we have chosen proteins with folding rate data available. This dataset has somewhat different structural alignment statistics than that for a non-redundant (NR) database, e.g. [105]. The TM-score based alignment of Zhang and Skolnick [106] can be used to obtain structural alignment statistics. Their method resolves the problems of outlier and length-dependent artifacts of RMSD-based alignments. Distributions of TM-score for both the above NR database, our dataset, and the datasets in references [96], [97], which non-knotted proteins in our dataset were taken, are given in figure SB of File S1, along with statistical analysis of the distributions. The bulk of our proteins (98%) have TM-scores consistent with the NR database of Thiruv *et. al* (see figure SB in File S1), however our dataset and those of [96], [97] contain a small number of structural homologs not present in the NR dataset, which are tabulated in table SB of File S1. We do not suspect that this small number of homologs will significantly modify the conclusions derived from statistical analysis of our dataset, however expansion and refinement to find the most relevant dataset is a topic for future work.

### Calculating distance metrics for the unfolded ensemble

To obtain minimal transformations between unfolded and native structures for a given protein, the 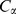 backbone was extracted from the PDB native structure, and 200 coarse-grained unfolded structures were generated using the methods described above. The unfolded structures were then aligned using RMSD and the average (residual) RMSD was calculated. The unfolded structures were then aligned by minimizing MRSD, and the residual MRSD was calculated. Then conformations were further coarse-grained (smoothed) by sampling every other bead, hence reducing the total number of beads. By the above further-coarse graining which is in the spirit of the initial steps of Koniaris-Muthukumar-Taylor reduction [107]–[110], we eliminate all instances of potential self-crossing in which the loop size or elbow size is smaller than three links. Each structure was then transformed to the folded state by the algorithm discussed earlier in Methods. The self-crossing instances, along with the coordinates of all the beads, were recorded as well. Appropriate data structures were formed and relevant crossing substructures (leg, elbow, and loop) were detected. With topological data structures at hand, the minimal uncrossing cost was found, through the depth-first search in the tree of possible uncrossing operations that was described above. Finally, the minimal uncrossing cost, 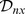, and the total distance, 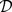 are calculated for each unfolded conformation. These differ from one unfolded conformation to the other; the ensemble average is recorded and used below. The ensemble average of MRSD and RMSD are also calculated from the 200 unfolded structures that were generated.

#### Importance of non-crossing

We define the importance of non-crossing (INX) as the ratio of the extra untangling movement caused by non-crossing constraints, divided by the distance when no such constraints exists, i.e. if the chain behaved as a ghost chain. Mathematically this ratio is defined as 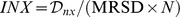.

#### Other metrics

Other metrics investigated include absolute contact order ACO [85], relative contact order RCO [85], long-range order LRO [86], and chain length N [87], [88].

Following [86], we define Long-range Order (LRO) as:
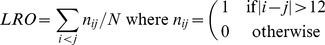
(4)where i and j are the sequence indices for two residues for which the 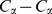 distance is 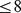 Å in the native structure.

Likewise we define Relative Contact Order (RCO) following [85]:
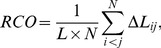
(5)where 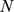 is the total number of contacts between non-hydrogen atoms in the protein that are within 6 Å in the native structure, 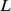 is the number of residues, and 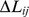 is the sequence separation between contacts in units of the number of residues.

Similarly, Absolute Contact order (ACO) [85] is defined to be:
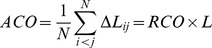
(6)


## Results

Proteins were classified by several criteria:

- 2-state vs. 3-state folders

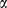
-helix dominated, vs 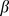-sheet dominated, vs mixed.
- knotted vs unknotted proteins

Several questions are answered for each group of proteins:

- What fraction of the total transformation distance is due to non-crossing constraints?
- How do the different order parameters distinguish between the different classes of proteins?
- How do the different order parameters correlate with each other?

### Order paramaters discriminate protein classes

In Table 2, we compare the unfolded ensemble-average of several metrics between different classes of proteins, and perform a p-value analysis based on the Welch t-test. The null hypothesis states that the two samples being compared come from normal distributions that have the same means but possibly different variances. Metrics compared in Table 2 are INX, LRO, RCO, ACO, MRDS, RMSD, 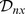, 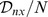, 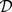, 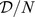 and 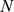.

**Table 2:** Comparison of order parameters for various protein classes.

Class	INX	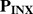	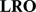	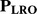	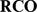	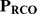
2-state folders	7.55e-02	(3.93e-01)	2.7	(9.46e-01)	1.58e-01	(5.07e-02)
3-state folders	8.25e-02		2.6		1.31e-01	
*α*-helix proteins	5.21e-02	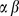 :4.01e-05	1.2	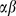 :7.40e-08	1.10e-01	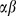 :3.34e-07
*β*-sheet proteins	9.04e-02	*β* m:(5.71e-01)	3.3	*β* m:(4.27e-01)	1.72e-01	*β* m:(2.68e-01)
Mixed secondary structure	8.64e-02	*α* m:5.44e-04	3.1	*α* m:6.20e-07	1.56e-01	*α* m:3.48e-03
Unknotted proteins	7.79e-02	1.48e-03	2.6	(9.20e-01)	1.49e-01	1.49e-02
knotted proteins	1.30e-01		2.7		1.24e-01	

Order parameters for various classifications of proteins. The data set of 2- and 3-state folders is the same as the data set for 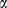-helical 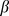-sheet and mixed proteins, and is given in Table 1. This is also the same data set as the unknotted proteins. Knotted proteins are separately classified, and not included as either 2-state or 3-state proteins. A discrimination is deemed statistically significant if the probability of the null hypothesis is less than 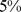.

The most obvious check of the general method outlined in the present paper is to compare the non-crossing distance 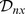 between knotted and unknotted proteins. Here we see that knotted proteins traverse about 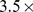 the distance as unknotted proteins in avoiding crossings, so that the two classes of proteins are different by this metric. The same conclusion holds for knotted *vs.* unknotted proteins if we use 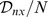, 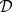, 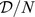, or INX. Of all metrics, the statistical significance is highest when comparing 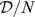, which is important because the knotted proteins considered here tend to be significantly longer than the unknotted proteins, so that chain length 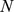 distinguishes the two classes. Dividing by 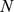 partially normalizes the chain-length dependence of 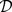, however 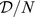 still correlates remarkably strongly with 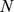 when compared for all proteins (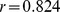 see table SJ in File S1).

It was somewhat unusual that MRSD and RMSD distinguished knotted proteins from unknotted proteins better than 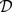 (or 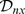), which accounts for non-crossing. All other quantities, including INX, ACO, and RCO distinguish knotted from unknotted proteins. The only quantity that fails is LRO.

The importance of noncrossing 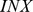, measuring the ratio of the uncrossing distance 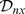 to the ghost-chain distance 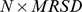, was largest for knotted proteins, followed by 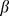 proteins, with 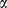 proteins having the smallest 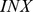. Mixed proteins had an average INX value in between that for 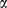 and 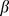 proteins.

In distinguishing all-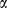 and all-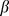 proteins, we find that LRO and RCO are by far the best discriminants. Interestingly, INX and 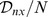 also discriminate these two classes comparably or better than ACO does. 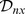 is marginal, while all other metrics fail.

All metrics except for 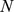 and 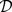 are able to discriminate 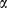 from mixed 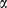-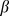 proteins, with LRO performing the best by far. Interestingly, none of the above metrics can distinguish 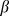 proteins from mixed 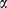-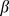 proteins.

It is sensible that energetic considerations would be the dominant distinguishing mechanism between two- and three state folders. Intermediates are typically stabilized energetically. We can nevertheless investigate whether any geometrical quantities discriminates the two classes. Indeed LRO and RCO fail, as does INX. This supports the notion that intermediates are not governed by “topological traps” that are undone by uncrossing motion, but rather are energetically driven. ACO performs marginally. Three-state folders tend to be longer than 2-state folders, so that 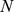 distinguishes them and in fact provides the strongest discriminant, consistent with previous results [111]. Interestingly RMSD, MRSD, and 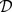 perform comparably to 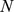. However these measures also correlate strongly with N (see table SJ in File S1). 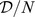, 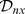 and 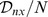 also perform well, but still correlate with N, albeit more weakly than the above metrics.

**Figure 15:**
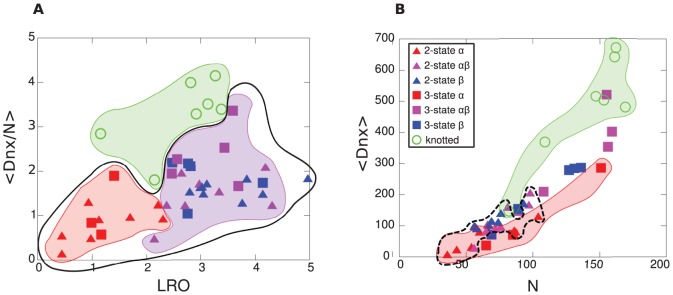
Clustering of protein classes depending on order parameter. (A) Scatter plot of all proteins as a function of 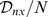 and 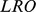. Knotted proteins are indicated as green circles and are clustered; unknotted proteins are clustered using with the black closed curve, and contain 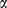-helical proteins clustered in red, and mixed 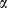-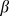 proteins clustered in magenta. Beta proteins are indicated in blue. Two and three state proteins are indicated as triangles and squares respectively. LRO provides a strong discriminant agains 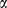 and mixed proteins, but not knotted and unknotted proteins, while 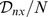 discriminates knotted from unknotted proteins, and moderately discriminates 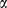 proteins from mixed proteins. (B) Scatter plot of all proteins as a function of 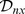 and 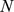. The rendering scheme for protein classes is the same as in panel (A). Kinetic 2-state folders are indicated by the black dashed curve. Both 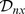 and 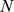 distinguish knotted from unknotted proteins, and 2-state from 3-state proteins. By projecting 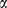 proteins and either mixed 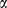/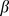 or all-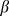 proteins onto each order parameter, one can see how 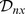 can discriminate 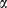 proteins from both mixed or 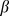 proteins, while 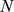 cannot. This is despite the significant correlation between 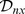 and 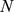.

Figure 15A shows a scatter plot of all proteins as a function of 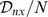 *vs.* and LRO. Knotted and unknotted proteins are indicated, as are 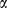, 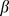, and mixed 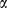-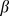 proteins. Two and three state proteins are indicated as triangles and squares respectively. From the figure, it is easy to visualize how LRO provides a successful discriminant between 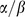 and 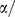
(mixed) proteins, but is unsuccessful in discriminating 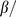
(mixed), knotted and unknotted, and two and three state folders. It is also clear from the figure how 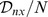 discriminates knotted from unknotted proteins. One can also see distribution overlap, but nevertheless successful discrimination between 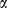 and 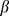 and 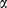 and mixed proteins.

Figure 15B shows a scatter plot of all proteins as a function of 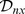 *vs.* 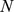, using the same rendering scheme for protein classes as in Figure 15A. From the figure, one can see how the metrics correlate with each other, and how they both discriminate knotted from unknotted proteins and 2-state from 3-state proteins. Moreover one can see how despite the significant correlation between 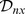 and 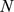, 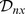 can discriminate 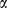 proteins from either 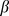 proteins or mixed 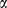/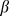 proteins, while N cannot.

As a control study for the above metrics, we took random selections of half of the proteins, to see if random partitioning of the proteins into two classes resulted in any of the metrics distinguishing the two sets with statistical significance. No metric in this study had significance: the p-values ranged from about 0.32 to 0.94.

**Figure 16:**
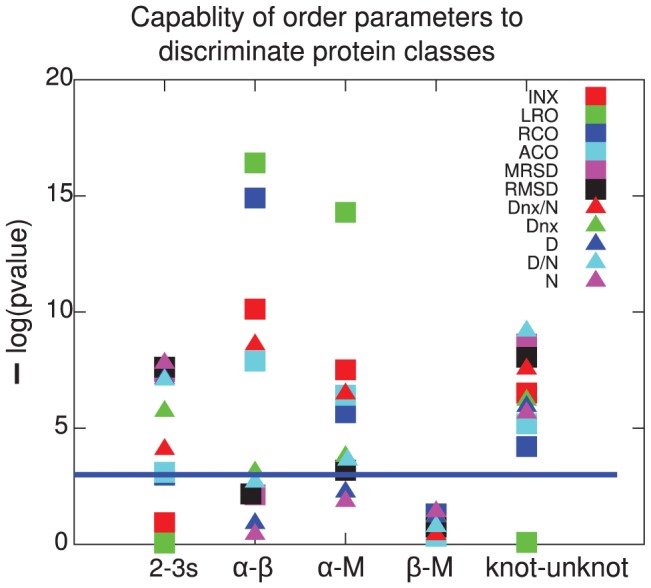
Statistical significance for all order parameters in distinguishing between different classes of proteins. The -log of the statistical significance is plotted as a function of pairs of protein classes, so that a higher number indicates better ability to distinguish between different classes. The blue horizontal line indicates a threshold of 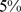 for statistical significance.

Figure 16 shows a plot of the statistical significance for all the metrics in Table 2 to distinguish various pairs of proteins classes: 2-state from 3-state proteins, 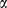 from 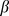, 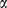 from mixed 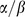, 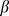 from mixed, and knotted from unknotted. We can define the most consistent discriminator between protein classes as that metric that is statistically significant for the most classes, and for those classes has the highest statistical significance. By this criterion 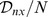 is the most consistent discriminator between the general structural and kinetic classes considered here.

Interestingly, in all cases, the extra distance introduced by non-crossing constraints is a very small fraction (less than 13%) of the MRSD, which represents the ghost distance neglecting non-crossing. This was not an obvious result, but it was encouraging evidence for the reason simple order-parameters that neglect an explicit accounting of non-crossing have been so successful historically [49], [85], [112]–[116].

### Scaling laws for pathway distances across domains and whole proteins

**Figure 17:**
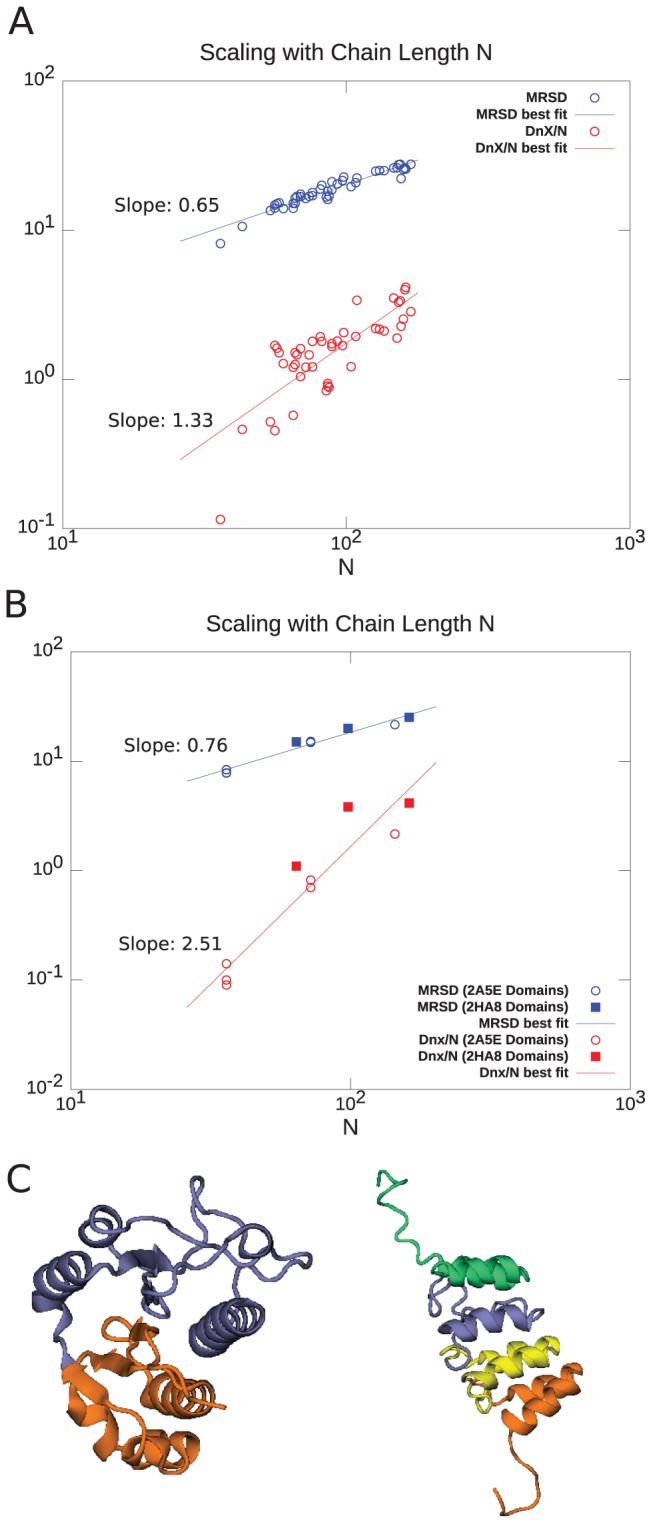
Approximate scaling laws for MRSD and non-crossing distance per residue 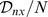
 **, across proteins and for domains within a single protein**. (A) MRSD (blue circles) as a function of chain length 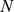 for our protein dataset. The slope of the best fit line on the log-log plot gives the power law scaling: 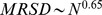. Non-crossing distance per residue 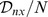 (red circles) *vs.* 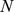 shows much larger scatter across native topologies, but follows an approximate scaling law 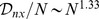 which is superextensive, indicating an increasing importance of chain non-crossing per residue as system size is increased. At system sizes larger than 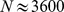, even minimal motion is dominated by entanglement. (B) Same quantities as in panel (A), but for the domains in proteins 2A5E and 2HA8. The scaling laws are different than in panel (A), and show stronger chain-length dependence. For 2HA8, domains 1, 2 and 1-2 together (the full protein) are considered; for 2A5E domains 1,2,3,4, 1-2, 2-3, 3-4, 1-2-3, 2-3-4, and 1-2-3-4 (the full protein) are considered. Based on these scaling laws found by building up proteins from subdomains, at system sizes larger than 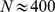, minimal pathways become entanglement-dominated. (C) Schematic renderings of the domains, color-coded in 2HA8 (left) and 2A5E (right).

Larger proteins will typically have larger MRSD. A protein of twice the chain length need not have twice the MRSD however; we plot the unfolded ensemble averaged MRSD of the proteins in our dataset as a function of 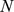 in Figure 17A. The plot shows sub-extensive scaling for the straight-line path distance per residue: 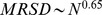. On the other hand, the non-crossing distance per residue, 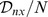, shows superextensive scaling: 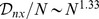, indicating that non-crossing induced entanglement becomes progressively more important even on a per-residue basis for longer proteins, and likely polymers in general. In fact, the steeper slope of 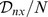 indicates a crossover such that when 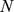 is larger than about 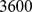, chain non-crossing dominates the motion of the minimal folding pathway. It is noteworthy that the scatter in the log-log scaling plot of Figure 17A is much larger for 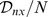 than for MRSD, illustrating the larger dispersion of 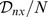 for proteins of the same length but different native topology.

The above analysis can be applied to domains within a single protein, to test how autonomous their folding mechanisms are as compared to separate proteins. Run on our dataset, the program DDomain [117] only finds multiple domains in methyltransferase domain of human tar (HIV-1) RNA binding protein (PDB 2HA8) (Wu, H. *et. al.* unpublished data), between residues 20–88 and 89–178 (residue 20 is the first resolved residue in the crystal structure). The domain finding program DHcL [118] also finds domains in this protein between residues 20–83 and 84–178. DHcL also finds domains in several other proteins, some generally accepted as single domain, however one of these proteins is clearly a repeat protein containing a 36 residue helix-turn-helix motif: tumor suppressor P16INK4A (PDB 2A5E) [119]. For this protein, DHcL finds domains between the 1st and 2nd, and 2nd and 3rd repeating units. We manually added a domain boundary between the 3rd and 4th repeating units to yield 4 domains containing residues 1–36, 37–72, 73–108, and 109–144. The domains of 2HA8 and 2A5E are illustrated in Figure 17C.

Using the above domain structures for 2HA8 and 2A5E, we analyze the scaling of MRSD with chain length N in Figure 17B. In these plots the individual domains are considered as separate proteins, then combined together if the domains are contiguous, e.g. for 2A5E proteins consisting of domains 1, domains 1 and 2 together, 1, 2, and 3 together, all domains together, and all contiguous combinations therein are examined. This yields the same scaling law for both proteins: 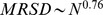, which has a larger power law than the scaling between proteins above. Chain connectivity constraints apparently induce cross-talk between domains even for MRSD. Likewise, the scaling law for noncrossing distance per residue is 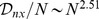, indicating significant polymer chain interference between domain folding. The individual domains of multidomain proteins apparently show less severe chain constraints than single domain proteins of the same size.

### Quantifying minimal folding pathways

The minimum folding pathway gives the most direct way that an unfolded protein conformation can transform by reconfiguration to the native structure. However, different configurations in the unfolded ensemble transform by different sequences of events, for example one unfolded conformation may require a leg uncrossing move, followed by a Reidemeister move elsewhere on the chain, followed by an uncrossing move of the opposite leg, while another unfolded conformation may require only a single leg uncrossing move.

**Figure 18:**
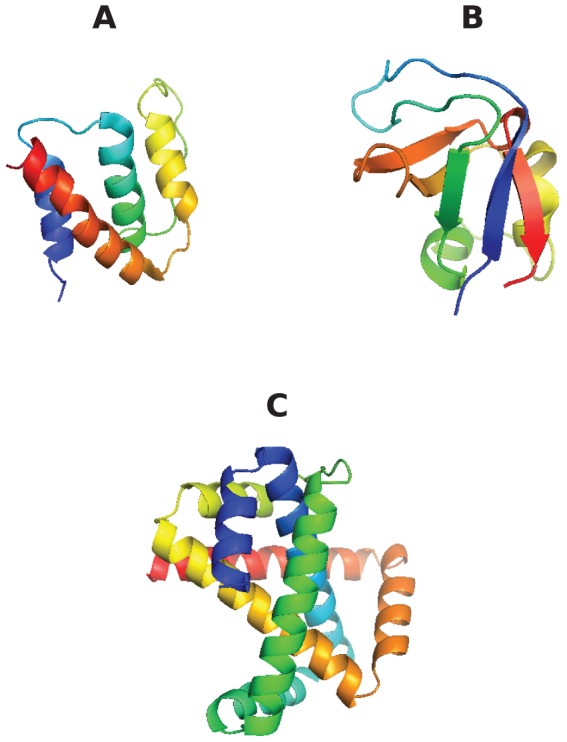
Schematic renderings of the three proteins whose minimal transformations we investigate in detail. (A) acyl-coenzyme A binding protein, PDB id 2ABD [120], an all-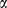 protein; (B) Src homology 3 (SH3) domain of phosphatidylinositol 3-kinase, PDB id 1PKS [121], a largely 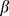 protein; (C) The designed knotted protein 2ouf-knot, PDB id 3MLG [100].

**Figure 19:**
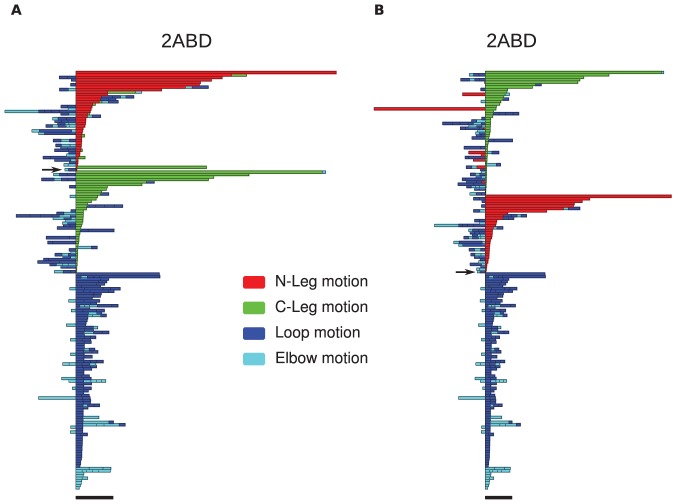
Bar plots for the uncrossing operations involved in minimal transformations from an unfolded ensemble, for the 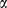 protein 2ABD. The sequence of noncrossing operations the transformation corresponding to a given pair of conformations is represented as a color-coded series of bars, with the sequence of moves going from right to left, and the length of the bar indicating the non-crossing distance undertaken by a particular move. Red bars indicate N-terminal leg (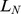) uncrossing, green bars indicate C-terminal leg (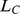) uncrossing, blue bars indicate Reidemeister “pinch and twist” loop uncrossing moves, and cyan bars indicate elbow uncrossing moves. The same set of 172 transformations is shown in panels A and B. Panel A sorts uncrossing transformations by rank ordering the following move types, largest to smallest: 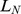, 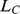, loop uncrossing, elbow move. Panel B sorts moves by 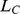, 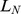, loop uncrossing, elbow move. The scale bar underneath each panel indicates a distance of 100 in units of the link length. The arrow in each panel denotes the “most representative” transformation, as defined in the text.

**Figure 20:**
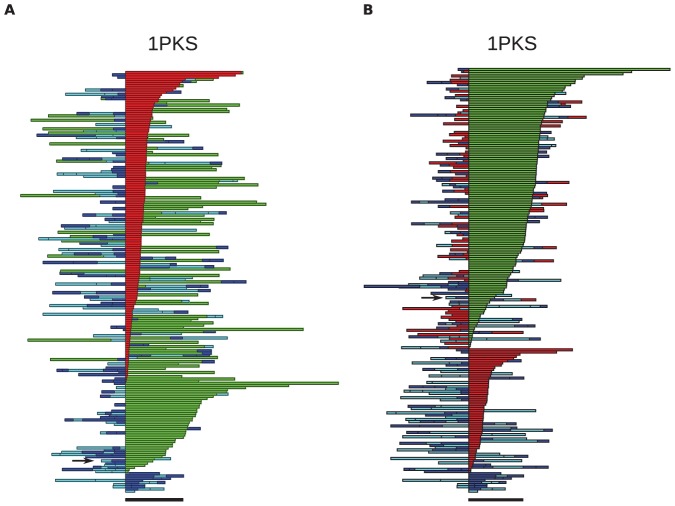
Bar plots of the uncrossing operations involved in minimal transformations for the 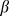-sheet protein 1PKS. See Figure 19 and the text for more details. Red bars: 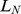 uncrossing moves; green bars: 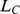 uncrossing moves; Blue bars: loop uncrossing moves; Cyan bars: elbow uncrossing moves. The same set of 195 transformations is shown in panels A and B, sorted as in Figure 19. The scale bar underneath each panel indicates a distance of 100 in units of the link length.

**Figure 21:**
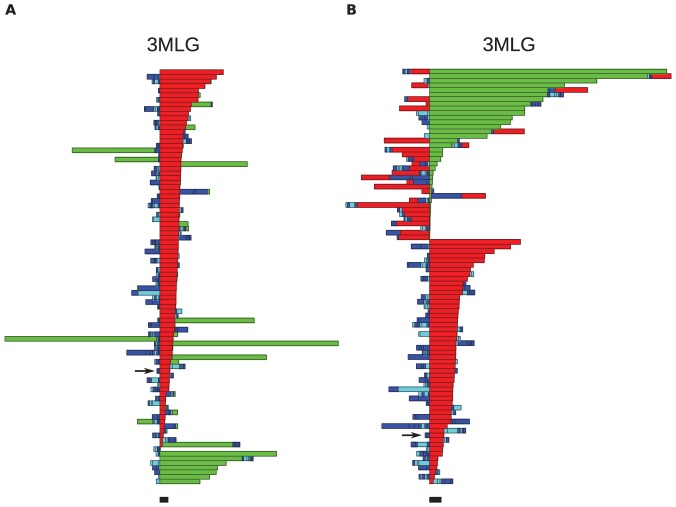
Bar plots of the uncrossing operations involved in the minimal transformations for the knotted protein 3MLG. See Figure 19 and the text for more details. Red bars: 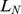 uncrossing moves; green bars: 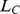 uncrossing moves; Blue bars: loop uncrossing moves; Cyan bars: elbow uncrossing moves. The same set of 90 transformations is shown in panels A and B, sorted as in Figure 19. The scale bar underneath each panel indicates a distance of 100 in units of the link length. The arrow in each panel denotes the “most representative” transformation, as defined in the text. The transformation located 8 bars up from the bottom of Panel A requires both 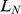 and 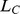 moves, however both leg motions are very small.

The sequence of moves can be represented as a color-coded bar plot, which, for the 3 proteins rendered in Figure 18, is shown in Figures 19, 20, 21. In these figures, the sequence of moves is taken from right to left, and the width of the bar indicates the non-crossing distance undertaken by that move. A scale bar is given underneath each figure indicating a distance of 100 in units of the link length. Red bars indicate moves corresponding to the N-terminal leg (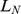) of the protein, while green bars indicate moves corresponding to the C-terminal leg (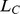). Blue bars indicate Reidemeister “pinch and twist” moves, while cyan bars indicate elbow uncrossing moves.

The typical sequence of moves varies depending on the protein. Figure 19 shows the uncrossing transformations of the all-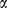 protein acyl-coenzyme A binding protein (PDB id 2ABD [120], see Figure 18A). Panels A and B depict the same set of transformations, but in A they are sorted from largest to smallest values of 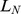 uncrossing, and in B they are sorted from largest to smallest values of 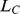 uncrossing. The leg moves in each panel are aligned so that the left end of the bars corresponding to the moves being sorted are all lined up. Some transformations partway down in panel A do not require an 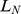 move; these are then ordered from largest to smallest 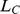 move. The converse is applied in panel B. Some moves do not require either leg move; these are sorted in decreasing order of the total distance of Reidemeister loop twist moves. Finally, some transformations require only elbow moves; these are sorted from largest to smallest total uncrossing distance.

Figure 20 shows the noncrossing transformations for the Src homology 3 (SH3) domain of phosphatidylinositol 3-kinase (PI3K), a largely-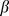 protein (about 23% helix, including 3 short 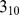 helical turns; PDB id 1PKS [121], see Figure 18B), sorted analogously to Figure 19. Figure 21 shows the uncrossing transformations involved in the minimal folding of the designed knotted protein 2ouf-knot (PDB id 3MLG [100], Figure 18C).

Interestingly, for the all-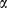 protein 2ABD, 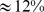 of the 172 transformations considered did not require any uncrossing moves, and proceed directly from the unfolded to the folded conformation. These transformations are not shown in Figure 19. For the 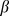 protein and knotted protein, every transformation that we considered (195 for 1PKS and 90 for 3MLG) required at least one uncrossing move.

As a specific example, the top-most move in Figure 21 panel B consists of a C-leg move (green) covering 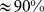 of the non-crossing distance, followed by N-leg move (red) covering 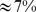 of the distance, then a short elbow move (cyan), a short Reidemeister loop move (blue), another short elbow move (cyan), and finally a short Reidemeister move (blue). In some cases the elbow and loop moves commute if they involve different parts of the chain, but generally they do not. For this reason we have not made any attempt to cluster loop and elbow moves, rather we have just represented them in the order they occur. On the other hand, consecutive leg moves commute and can be taken in either order.

In Figures 19, 20,21, one can see that significantly more motion is involved in the leg uncrossing moves than for other types of move. The total distance covered by leg moves is 82% for 3MLG, 69% for 1PKS, and 49% for 2ABD. For 3MLG, the total leg move distance is comprised of 44% 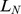 moves, and 38% 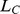 moves. For 1PKS, leg move distance is comprised of 18% 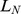 moves, and 51% 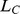 moves. For 2ABD, distance for the leg moves is roughly symmetric with 26% 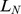 and 23% 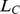.

One difference that can be seen for the all-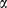 protein compared to the 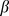 and knotted proteins is in the persistence of the leg motion. For 2ABD, only 24% of the transformations require 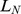 moves and only 30% of the transformations require 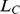 moves. On the other hand the persistence of leg moves is greater in the 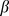 protein and greatest in the knotted protein. For 1PKS, 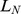 and 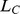 moves persist in 74% and 66% of the transformations respectively. In 3MLG, 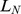 and 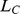 moves persist in 92% and 41% of the transformations respectively.

Inspection of the transformations for the 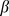 protein 1PKS in panels A and B of Figure 20 reveals that uncrossing moves generally cover larger distance than in the 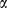 protein 2ABD (the mean uncrossing distance for is 136 for 1PKS *vs.* 77.5 for 2ABD). We also notice that in contrast to the leg uncrossing moves in 2ABD, both 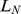 and 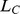 moves are often required (44% of the transformations require both 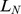 and 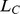 moves, compared to 5% for 2ABD). The asymmetry of the protein is manifested in the asymmetry of the leg move distance: the 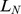 moves are generally shorter than the 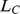 moves, covering about 1/4 of the total leg move distance. As mentioned above, 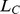 moves comprise about 51% of the total distance for the 195 transformations in 20, while 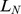 moves only comprise about 18% of the distance on average. Both 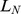 and 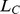 moves are persistent as mentioned above. A leg move of either type is present in 95% of the transformations.

Inspection of the transformations in Figure 21 reveals that every transformations requires either an 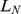 or 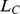 move. This is sensible for a knotted protein, and is in contrast to the transformations for the 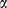 protein 2ABD, where many moves do not require any leg uncrossing at all and consist of only short Reidemeister loop and elbow moves. In this sense the diversity of folding routes [40], [41] for the knotted protein 3MLG is the smallest of the proteins considered here, and illustrates the concept that topological constraints induce a pathway-like aspect to the folding mechanism. The N-terminal 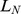 leg move is the most persistently required uncrossing move, present in about 92% of the transformations. This is generally the terminal end of the protein that we found was involved in forming the pseudo-trefoil knot. Sometimes however, the C-terminal end is involved in forming the knot, though this move is less persistent and is present in only 41% of the transformations. However when an 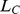 move is undertaken, the distance traversed is significantly greater, as shown in Panel B of Figure 21. This asymmetry is a consequence of the asymmetry already present in the native structure of the protein.

### Consensus minimal folding pathways

**Figure 22:**
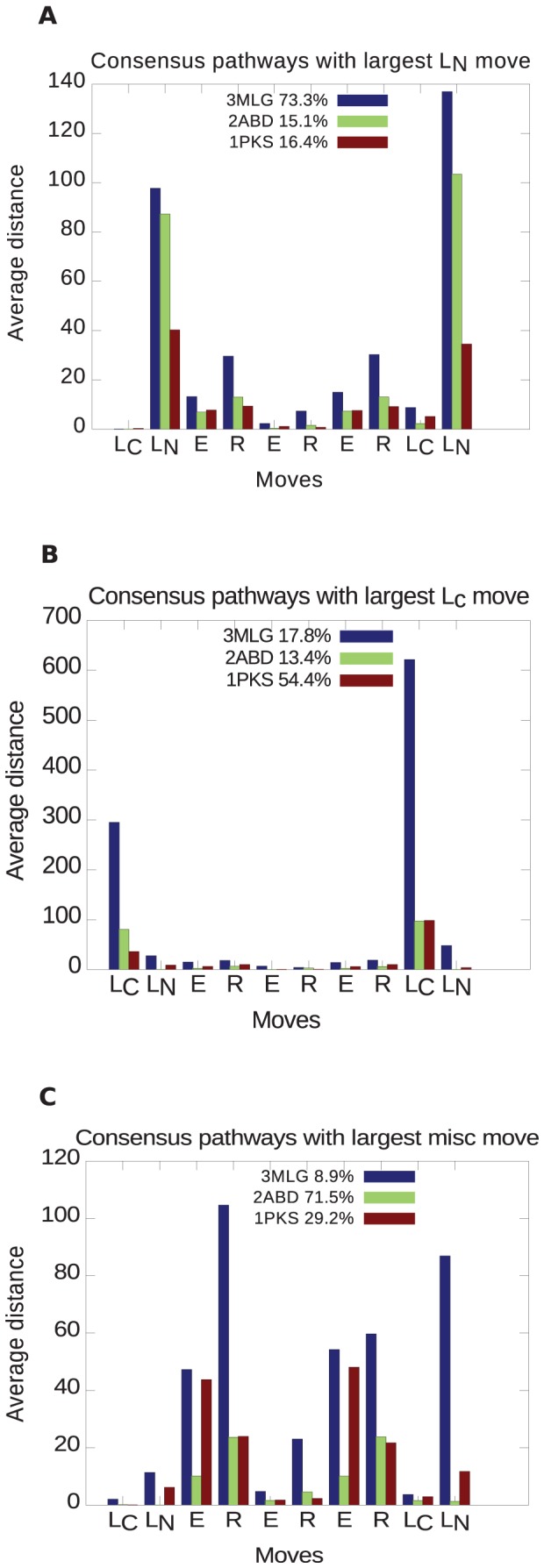
Consensus histograms of the transformations described in Figures 19, 20, 21. See text for a description of the construction. Each bar represents the distance of a corresponding move type, N or C leg (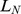 or 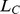), elbow 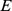, or loop 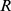. The order of the sequence of moves is taken from right to left along the x-axis. An all-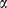 protein (2ABD), an all-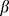 protein (1PKS), and a knotted protein (3MLG) are considered. (A) Transformations with leg 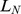 as the largest move. These encompass 15% of the transformations those in the 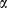 protein, 16% of the transformations in the 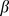 protein, and 73% of the transformations for the knotted protein. (B) Transformations with leg 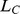 as the largest move, which encompass 13% of the 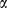 protein transformations, 54% of 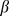 protein transformations, and 18% of knotted protein transformations. (C) Transformations with either an elbow E or loop R as the largest move, which encompass 71% of the 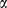 protein transformations, 29% of 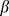 protein transformations, and 9% of knotted protein transformations.

From the transformations described in Figures 19, 20, 21, we see that there are a multitude of different transformations that can fold each protein. The pathways for the 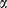 protein 2ABD are more diverse than those for the 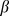 or knotted proteins. From the ensemble of transformations for each protein, we can average the amount of motion for each uncrossing move to obtain a quantity representing the consensus or most representative minimal folding pathway for that protein. This takes the form of the histograms in Figure 22, with the x-axes representing the order of uncrossing/untangling events, right to left, and the y-axes representing the average amount of motion in each type of move.

The ensemble of untangling transformations can be divided into three different classes: transformations in which leg 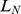 is the largest move, transformations in which leg 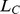 is the largest move, and transformations in which an elbow E or loop R (for Reidemeister type I) are the largest moves. Moreover, if 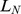 and 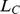 moves occur consecutively they can be commuted, so without loss of generality we take the 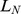 move as occurring before the 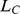 move in the x-axes of Figure 22. The leg moves, if they occur first, are then followed by either elbow (E) and/or loop (R) moves, of which there may be several. In general, the leg moves may both occur before the collection of loop and elbow moves, after them, or may bracket the elbow and loop moves (e.g. 2nd bar in Figure 21b). By the construction of our approximate algorithm, if two 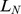 moves were encountered during a trajectory (they were encountered only a few times during the course of our studies), they would be aggregated into one 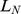 move involving the larger of the two motions, in order to remove any possible redundancy of motion. Hence no more than one 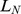 or 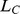 move is obtained for all transformations. We found that three pairs of elbow and loop moves was sufficient to describe about 93% of all transformations (see the x-axes of Figure 22). In summary, the sequence 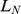, 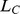, 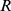, 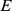, 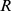, 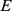, 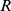, 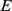, 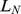, 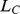 (read from left to right) characterized almost all transformations, and so was adopted as a general scheme. Any exceptions simply had more small elbow and loop moves that were of minor consequence; for these transformations we simply accumulated the extra elbow and loop moves into the most appropriate 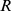 or 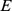 move. The general recipe for rendering loops R in Figure 22 is as follows: if one R move is encountered (regardless of where), each half is placed first and last (third) in the general scheme. If two R moves are encountered, they are placed first and last, and if three R moves are encountered, they are simply partitioned in the order they occurred. For four or more R moves, the middle 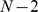 are accumulated into the middle slot in the general scheme. The same recipe is applied to elbow moves E. As a specific example, the first bar in Figure 21b consists of 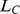, 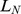, 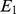, 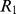, 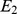, 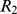, which after permutation of the first two leg moves falls into the general scheme above as 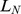, 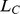, 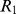, 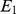, 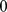, 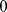, 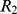, 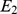, 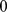, 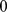. The bottom-most transformation in Figure 21B consists of 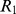, 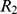, 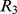, 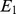, 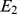, 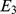, 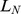, which becomes 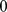, 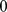, 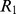, 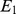, 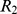, 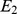, 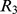, 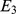, 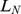, 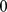 in the general scheme.

Figure 22 shows histograms of the minimal folding mechanisms, obtained from the above-described procedure. Note again there are 3 classes of transformation, one where 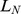 is the largest move, one where 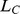 is the largest move, and one where either loop R or elbow E is the largest move. Each uncrossing element of the transformation, C-leg, N-leg, Reidemeister loop, or elbow, contributes to the height of the corresponding bar, which represents the average over transformations in that class. The percentage of transformations that fall into each class is given in the legend to panels A–C of Figure 22.

Most of the transformations (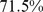) for the 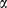-protein 2ABD fall into the class with a dominant loop or elbow move, which itself tends to cover less uncrossing distance than either C- or N-leg uncrossing (ordinates of Panels A–C Figure 22). This is a signature of a diverse range of folding pathways: minimal folding pathways need not involve obligatory leg uncrossing constraints. In this sense, the 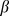 protein 1PKS is more has a more constrained folding mechanism than the 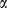 protein; there is a significantly larger percentage of transformations for which a leg transformation 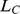 or 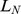 dominates, though the mean distances undertaken when a leg move does dominate are comparable for 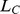 and even larger for the 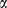 protein for 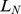.

The knotted protein 3MLG has the most constrained minimal folding pathway. A leg move from either end dominates for 91% of the cases. Even for the transformations where loop or elbow moves dominate, there is still relatively significant 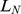 motion. The dominant pathways for knotting 3MLG involve leg crossing from either N or C terminus. When the C terminus is involved in the minimal transformation, the motion can be significant (Figure 22B).

**Figure 23:**
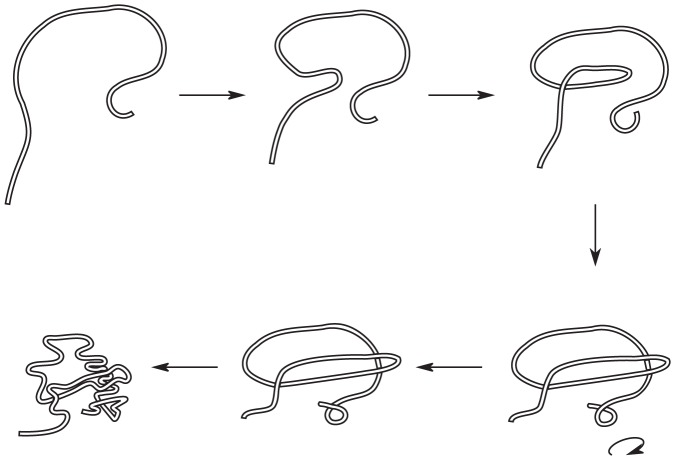
Schematic of the most representative transformation for the 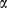 protein 2ABD.

**Figure 24:**
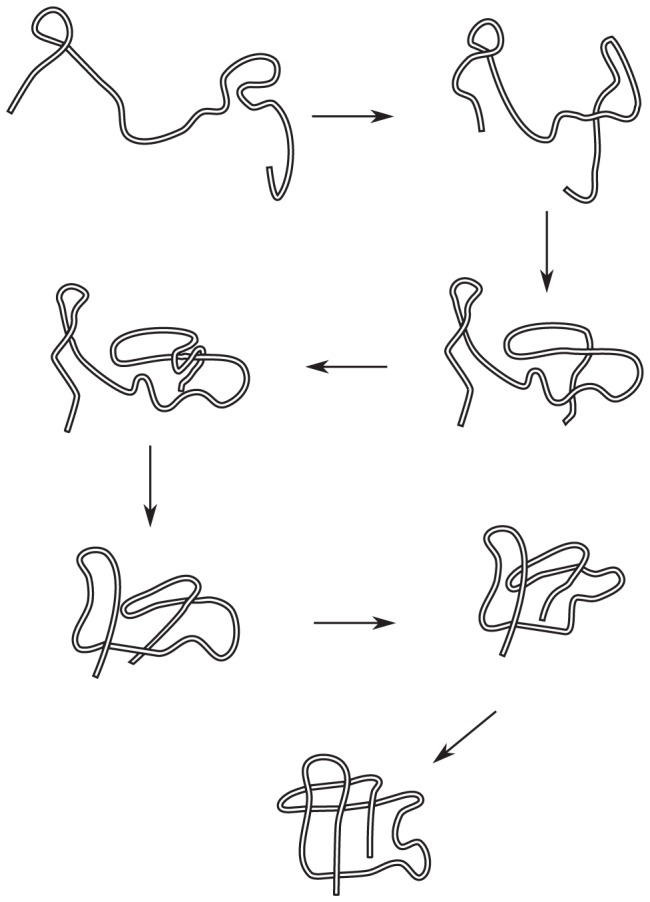
Schematic of the most representative transformation for the 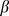 protein 1PKS.

**Figure 25:**
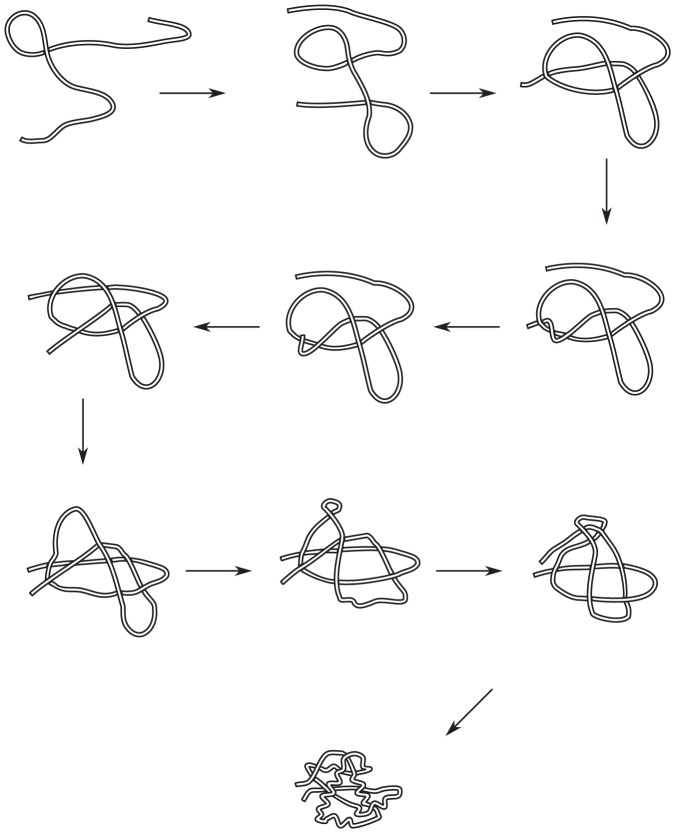
Schematic of the most representative transformation for the knotted protein 3MLG.

Among all transformations of a given protein, a transformation can be found that is closest to the average transformation for one of the three classes in Figure 22. This consensus transformation has a sequence of moves that when mapped to the scheme in Figure 22, has minimal deviations from the averages shown there. Further, we can find the transformation that has minimal deviation to any of the three classes in Figure 22. For the knotted protein 3MLG, the best fit transformation is to the class with 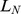-dominated moves, for the 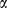 protein 2ABD, the best fit transformation is to the class with miscellaneous-dominated moves, and the 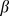 protein 1PKS, the best fit transformation is the class with 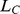-dominated moves. For the 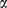, 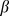, and knotted proteins, these are the transformations denoted by a short arrows to the left of the transformation in panels A and B of Figures 19, 20, and 21 respectively. For the 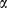, 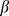, and knotted proteins, the transformations are illustrated schematically in Figures 23, 24, and 25 respectively.

Inspection of the most representative transformation for the all-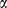 protein 2ABD shown in Figure 23 indicates that the transformation requires remarkably little motion: it contains a negligible leg motion followed by a loop uncrossing of modest distance, followed by a short elbow move that is also inconsequential: in shorthand 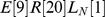, where the numbers in brackets indicate the cost of the moves in units where the link length is unity. In constructing a schematic of the representative transformation in Figure 23, we ignore the smaller leg and elbow moves and illustrate the loop move roughly to scale. Although additional crossing points appear from the perspective of the figure, the remainder of the transformation involves simple straight-line motion.

Figure 24 shows the most representative folding transformation for the 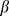 protein 1PKS. The sequence of events constructed from the most representative minimal transformation, 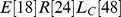, consists of a dominant leg move depicted in steps 4 and 5 of the transformation, followed by shorter loop and elbow Reidemeister moves that are neglected in the schematic. Loops and crossing points appear from the perspective of the figure, however the remainder of the transformation involves simple straight-line motion.

Figure 25 shows the most representative folding transformation for the knotted protein 3MLG. The sequence of events constructed from the minimal transformation, 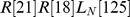 in the above notation, consists of a dominant leg move depicted in steps 4 and 5 of the transformation, and two relatively short loop moves that are neglected in the schematic as inconsequential. Loops appear from the perspective of the figure, and the crossing points appear to shift in position, however the remainder of the transformation involves simple straight-line motion.

### Topological constraints induce folding pathways

From Figures 19, 20, 21, one can see that topological non-crossing constraints can induce pathway-like folding mechanisms, particularly for knotted proteins, and in part for 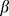-sheet proteins as well. The locality of interactions in conjunction with simple tertiary arrangement of helices in the 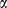-helical protein profoundly affects the nature of the transformations that fold the protein, such that the distribution of minimal folding pathways is diverse. Conversely, the knotted protein, although largely helical, has non-trivial tertiary arrangement, which is manifested in the persistence of a leg crossing move in the minimal folding pathway. In this way, a folding “mechanism” is induced by the geometry of the native structure.

We can quantify this notion by calculating the similarity between minimal folding pathways. To this end we note that, for example, the transformation that is 6 bars from the bottom in Figure 21b, which contains an 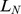 move followed by 2 short loops and an elbow, should not fundamentally be very different than the transformation 10 from the bottom in that figure, which contains a loop and 2 short elbows followed by a larger 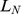 move. In general we treat the commonality of the moves as relevant to the overlap rather than the specific number of residues involved, or the order of the moves that arises from the depth-first tree search algorithm.

**Figure 26:**
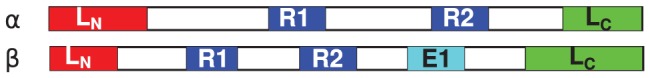
Overlap between minimal transformations. Schematic diagram for the residues involved in uncrossing operations for two minimal transformations labelled by 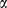 and 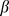, to illustrate the sequence overlap between transformations.

Thus for each transformation pair we define two sequence overlap vectors in the following way. Overlaying the residues involved in moves for each transformation along the primary sequence on top of each other as in Figure 26, we count as unity those moves of the same type that overlap in sequence for both transformations, otherwise a given move is assigned a value of zero. So for example in Figure 26 the result is two vectors of binary numbers, one with 4 elements for transformation 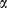 and one with 5 elements for transformation 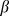, based on the overlap of moves of the same type. That is, the first vector is 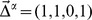 and the 2nd vector is 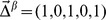. To find the pathway overlap, we also record the noncrossing distances of the various transformations which here would be two vectors of the form 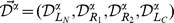, and 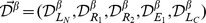. Square matrices 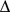 are constructed for 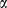 and 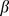, where each row is identical and equal to the vector 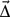. This matrix then operates on 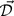 to make a new vector that has distances for the elements that are nonzero in 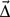, and is the same length for both 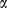 and 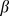. In the above example shown in Figure 26, 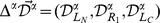 and 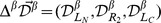. These vectors are then multiplied through the inner product, and divided by the norms of 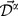 and 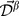 to obtain the overlap 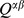. In the above example, 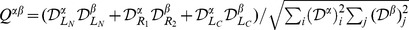. In general, the formula for the overlap is given by
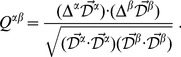
(7)When 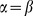, 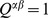. In the above example, 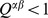 even if all loops were aligned, because there is no elbow move in transformation 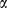. If two transformations have an identical set of moves, 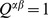 if all the moves have at least partial overlap with a move of the same type in primary sequence. If a loop move in transformation 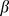 overlaps two loop moves in transformation 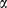, it is assigned to the loop with larger overlap in primary sequence. For the first two transformations in Figure 21A, 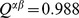, and for the first two transformations in Figure 21B, 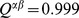. On the other hand for the first and last transformations in Figure 21B, 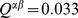.

**Figure 27:**
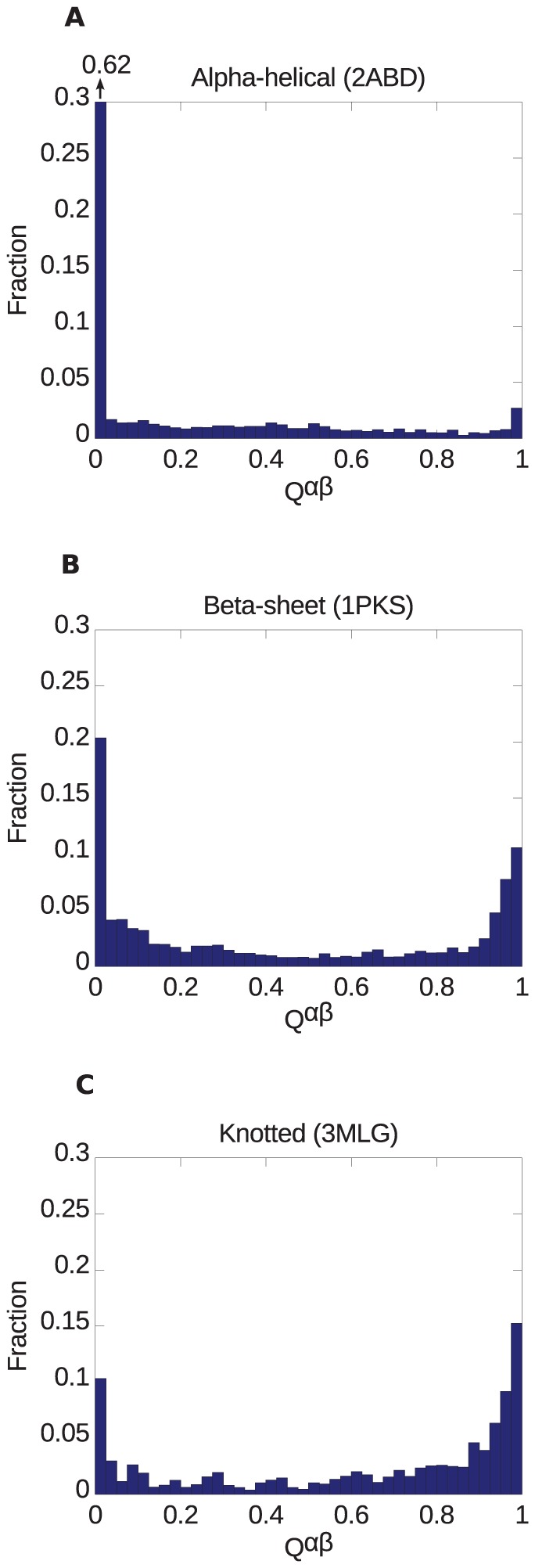
Distribution of pathway overlap between minimal transformations, for an 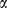, 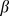, and knotted protein. Pathway overlap (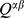) distributions for the 3 proteins in Figure 18, as defined by Equation (8), operating on the transformations in Figure 19, 20, 21. (a) The pathway overlap distribution for the all-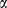 protein 2ABD indicates a large contribution for 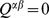 (the peak height in the distribution is 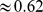), indicating a diverse set of minimal transformations fold the protein. The average 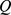 for these transformations is 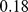. (b) The pathway overlap distribution for the 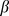-protein shows the emergence of a peak around 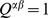, indicating partial restriction of folding pathways. The peak near 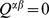 still carries more weight in the distribution. The average 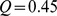. (c) The peak around 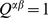 becomes dominant for the pathway overlap distribution of the knotted protein, indicating the emergence of a dominant restricted minimal folding pathway. The average 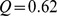.

Figure 27 shows the distributions of overlaps 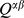 between all pairs of transformations indicated in Figures 19, 20, 21, for the three proteins shown in Figure 18. The distributions show a transition from multiple diverse minimal folding pathways for the 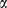 protein, to the emergence of a dominant minimal folding pathway for the knotted protein. The mean overlap 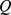 between transformations can be obtained by averaging 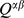 in Equation (8) over all pairs of transformations: 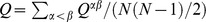. Mean overlaps for each protein are given in the caption to Figure 27. This illustrates that topological constraints induce mechanistic pathways in protein folding. We elaborate on this in the Discussion section.

## Discussion

The Euclidean distance between points can be generalized mathematically to find the distance between polymer curves; this can be used to find the minimal folding transformation of a protein. Here, we have developed a method for calculating approximately minimal transformations between unfolded and folded states that account for polymer non-crossing constraints. The extra motion due to non-crossing constraints was calculated retroactively for all crossing events of a ghost chain transformation involving straight line motion of all beads on a coarse-grained model chain containing every other C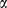 atom, from an ensemble of unfolded conformations, to the folded structure as defined by the coordinates in the protein databank archive. The distances undertaken by the uncrossing events correspond to straight-line motions of all the beads from the conformation before the crossing event, over and around the constraining polymer, and back to the essentially identical polymer conformation immediately after the crossing event. Given a set of chain crossing events, the various ways of undoing the crossings are explored using a depth-first tree search algorithm, and the transformation of least distance is recorded as the minimal transformation.

We found that knotted proteins quite sensibly must undergo more noncrossing motion to fold than unknotted proteins. We also find a similar conclusion for transformations between all-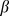 and all-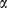 proteins; all-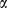 proteins generally undergo very little uncrossing motion during folding. In fact the uncrossing distance, 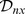, averaged over the unfolded ensemble, can be used as a discrimination measure between various structural and kinetic classes of proteins. Comparing several metrics arising from this work with several common metrics in the literature such as RMSD, absolute contact order ACO, and long range order LRO, we found that the most reliable discriminator between structural classes, as well as between two- and three-state proteins, was 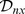 per residue. (later paragraph moved here:) Knotted proteins, as compared to unknotted proteins, are the most distinguishable class of those we investigated, in that all metrics we investigated except for LRO significantly differentiated the knotted from unknotted proteins. The differentiation between structural or kinetic classes of proteins as studied here is a separate issue from the question of which order parameters may best correlate with folding rates [28], [86], [96], [122], [123]; this latter question is an interesting topic of future research. Differentiating native-structure based order parameters that provide good correlates of folding kinetics is a complicated issue, in that different structural classes may correlate better or worse with a given order parameter [123].

Non-crossing distance per residue 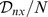 increases more rapidly with chain length than the mean straight line distance between residue pairs (MRSD). Considering proteins as separate domains indicates a crossover at long chain length, about 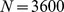. Considering proteins built up by adding successive domains, specifically for two representative multi-domain proteins in our dataset (2HA8 and 2A5E), indicates a crossover to entanglement-dominated folding mechanisms at shorter lengths- about 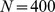. This crossover point may indicate a regime where energetics begins to play a role in order to fold domains independently, and avoid progressively more significant polymer disentanglement in order to fold.

Even for knotted proteins, the motion involved in avoiding non-crossing constraints is only about 13% of the total ghost chain motion undertaken had the noncrossing constraints been neglected. This was not an obvious result, to these authors at least. In contrast to melts of long polymers, chain non-crossing and the resultant entanglement does not appear to be a significant factor in protein folding, at least for the structures and ensembles we have studied here. It is tempting to conclude from this that chain non-crossing constraints play a minor role in determining folding mechanisms. It is nevertheless an empirical fact that knotted proteins fold significantly slower than unknotted proteins [100], [124]. As well, raw percentages of total motion do not take into account the difficulty in certain types of special polymer movement, in particular when the entropy of folding routes is tightly constrained [12], [40], [41], [125]–[127]. The small percentage of non-crossing motion may offer some explanation however, as to why simple order parameters, such as absolute contact order, that do not explicitly account for noncrossing in characterizing folding mechanisms have historically been so successful in predicting kinetics.

The non-crossing distance was calculated here for a chain of zero thickness, so that non-crossing is decoupled from steric constraints. Finite volume steric effects would likely enhance the importance of non-crossing constraints, since the volume of phase space where chains are non-overlapping is reduced, and thus chain motions must be further altered to respect these additional constraints [128]. Steric constraints may significantly alter the shape of reactive trajectories, and slow kinetics by enforcing entropic bottlenecks. Such constraints may become particularly important for collapsed or semi-collapsed proteins, and knotted proteins where they restrict stereochemically-allowed folding pathways. These effects may in principle be treated by extending the present formalism to include non-zero chain thickness, and by extending the minimal folding pathway to the partition function of pathways, with each pathway having weight proportional to the exponent of the distance [72]. Such a treatment is an interesting and important topic of future work.

One potential issue in the construction of the algorithm used here is that the approximated minimal transformation is generally not equivalent to a kinetically realizable transformation. In the depth-first tree search algorithm illustrated in Figure 14, the set of crossing points defines a set of uncrossing moves that may be permuted, or combined for example through a compound leg movement as in Figure 10. However the kinetic sequence of crossing events, in particular those significantly separated in “time” along the minimal transformation, may not be permutable or combinable physically, at least not without modifying the distance travelled [129] Hence the transformations are treated here as approximations to the true minimal transformations that respect non-crossing.

The algorithm as described above may underrepresent the amount of motion involved in noncrossing by allowing kinetically separated moves to be commutable. On the other hand, the motion assumed in the algorithm to be undertaken by a crossing event contains abrupt changes in the direction of the velocity (corners) at the time of the uncrossing event, and so is larger than the true minimal distance. These errors cancel at least in part. It is an interesting topic of future research to develop an improved algorithm that computes minimal transformations, perhaps using these approximate transformations as a starting point for further optimization or modification.

The mathematical construction of minimal folding transformations can elucidate folding pathways. To this end we have dissected the morphology of protein structure formation for several different native structures. We found that the folding transformations of knotted proteins, and to a lesser extent 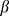 proteins, are dominated by persistent leg uncrossing moves, while 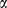 proteins have diverse folding pathways dominated simply by loop uncrossing.

A pathway overlap function can then be defined, the structure of which is fundamentally different for 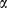 proteins than for knotted proteins. While the overlap function supports the notion of a diverse collection of folding pathways for the 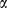 protein, the overlap function for the knotted protein indicates that topological polymer constraints can induce €mechanism€ into how a protein folds, i.e. these constraints induce a dominant sequence of events in the folding pathway. This effect is observed to some extent in the 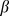 protein we investigated, but is most pronounced for knotted proteins.

Other approaches have been made previously to quantify topological frustration, and construct folding pathways that minimize such frustration. Norcross and Yeates [126] have extended the earlier analysis of Connolly *et. al* [130], to show that edges between consecutive 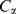 atoms in the coarse-grained primary sequence can be surrounded by a ring of other 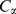 atoms consisting of the vertices of tetrahedra from Delauney tesselation. They then find the folding pathways that minimize the number of times a ring forms before its thread is formed within a single-sequence approximation: these indicate topologically-frustrated pathways. As an interesting example in [126], strand IV of superoxide dismutase (SOD1) is highly buried by parts of the Zn-binding loop, electrostatic loop, and neighboring strands V and VII. In vitro folding studies [131], [132] show however that this problem is resolved by Zn-binding after folding of the 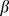-barrel, which is coupled with structural formation of the Zn-binding and electrostatic loops (loops IV and VII). The apo state is an energetically stressed, metastable intermediate [133]. In general, folding coupled to ligand binding could remove topological frustration by inducing unfrustrated pathways in the folding mechanism.

Similar schematic “average” folding mechanisms as in Figures 23, 24, 25, based on minimal folding pathways, were proposed for the complex Stevedore knotted protein 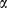-haloacid dehalogenase by Bölinger *et al* [102], based on folding simulation statistics of G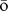 models.

Coarse-grained simulation studies of the reversible folder YibK [99] showed that non-native interactions between the C-terminal end and residues towards the middle of the sequence were a prerequisite for reliable folding to the trefoil knotted native conformation [134], the evolutionary origins of which were supported by hydrophobicity and 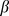-sheet propensity profiles of the SpoU methyltransferase family. This suggests a new aspect of evolutionary “design” involving selective non-native interactions, beyond the generic role that non-native interactions may play in accelerating folding rate [135], [136]. Low kinetic success rates 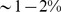 in purely structure-based Gō simulations are also seen in coarse-grained simulation studies of YibK [137] and all-atom simulation studies of the small 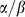 knotted protein MJ0366 [138]. In these studies by Onuchic and colleagues, a “slip-knotting” mechanism driven by native contacts is proposed, rather than the “plug” mechanism in [134], which is driven by non-native contacts. Both slip-knotting and plug mechanisms were described by Mohazab and Plotkin as optimal un-crossing motions of protein chains in [74]. Such mechanisms may be facilitated by flexibility in the protein backbone: highly conserved glycines in the hinge regions of both knotted and slipknotted [139] proteins modulate the knotted state of the corresponding subchain of the protein [140]. Further bioinformatic studies that investigate evolutionary selection by strengthening critical native or non-native interactions in knotted proteins are an interesting topic of current and future research. There is certainly a precedent of selection for native interactions that penalize on-pathway intermediates in some proteins such as ribosomal protein S6 [40], [41], [141]. Structural analysis of the deeply buried trefoil knot in acetohydroxy acid isomeroreductase indicates swapping of secondary structural elements across replicated domains likely arising from gene duplication [109], which argues in favor of knot formation driven by native interactions, through a mechanism apparently distinct from slipknotting.

Lua and Grosberg have found that, due to enhanced return probabilities originating from finite globule size along with secondary structural preferences, protein chains have smaller degree of interpenetration than collapsed random walks, and thus fewer knots than would be expected for such collapsed random walks [142], in spite of the fact that collapse dramatically enhances the likelihood of knot formation [110], an effect foreshadowed by the dramatic decrease in characteristic length for knot formation as solvent quality changes from good to ideal (theta) [107], [108]. It is still not definitively answered whether this statistical selection against knots in the protein universe is a cause or consequence of the above size and structural preferences. Similarly, Mansfield [143], [144] has suggested that the polar nature of the N- and C- termini of the protein chain energetically penalize processes that would result in the formation of knots.

Conversely, some functional roles may benefit from the presence of knotted topologies. Virnau and colleagues [145] have suggested that the presence of complex knots in proteins involved in regulation of ubiquitination and proteolysis serve a protective role against incidental proteasome degradation, and as well, they observe evidence for the modulation of function by alteration of an enzymatic binding site through either the presence or absence of a knot in homologues of transcarbamylase. Phylogenetic analysis indicates that the presence of a knot is most likely mediated by a single evolutionary event involving insertions of short segments in the primary sequence [146].

The interplay between sequence-determined energetics and chain connectivity in the folding of proteins with complex or knotted topologies is a topic of much current interest, despite the fact that the number of proteins exhibiting knots or slipknots in their native structures is relatively small. It will be interesting to see how evolution has optimized sequence or facilitated protein-chaperone interactions to enable folding for these “problem children” of the proteome.

## Supporting Information

Movie S1 **Approximate solution to minimal distance transformation from a vertical line to a horizontal one.**
(GIF)

Movie S2 **Approximate solution to minimal distance transformation from an unfolded conformation of protein 1CSP to the folded conformation, where the chains are ghost chains.**
(GIF)

Movie S3 **Approximate solution to minimal distance transformation from an unfolded conformation of protein 1CSP to the folded conformation, where the chains are ghost chains; instances of self-crossing are emphasized.**
(GIF)

File S1 Includes: Description and analysis showing that crossing detection and uncrossing distance are independent of the projection plane, including Figure SA; Structural alignment statistics of our protein dataset, including Table SA, Table SB, and Figure SB; Cross correlation tables of order parameters, Tables SC-SJ; References.
(PDF)
