# Supplementary figures and images for: Polymer Uncrossing and Knotting in Protein Folding, and Their Role in Minimal Folding Pathways
Source: PLoS One. 2013 Jan 24;8(1):e53642. doi: 10.1371/journal.pone.0053642 (PMC3554774; doi:10.1371/journal.pone.0053642)

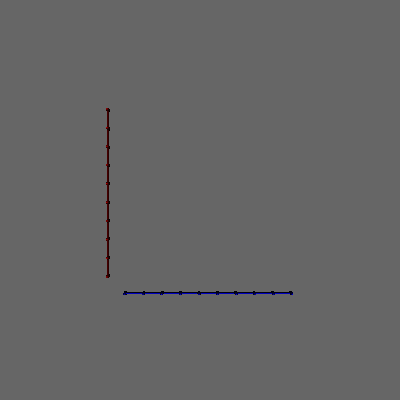

Supplement: Movie S1 — Approximate solution to minimal distance transformation from a vertical line to a horizontal one. (GIF) [file pone.0053642.s001.gif]

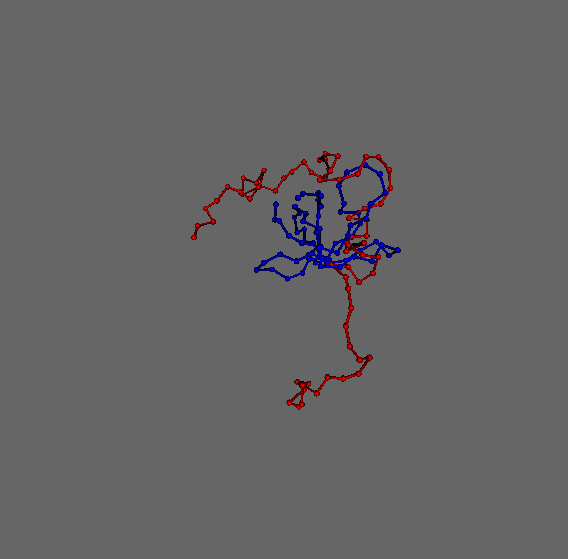

Supplement: Movie S2 — Approximate solution to minimal distance transformation from an unfolded conformation of protein 1CSP to the folded conformation, where the chains are ghost chains. (GIF) [file pone.0053642.s002.gif]

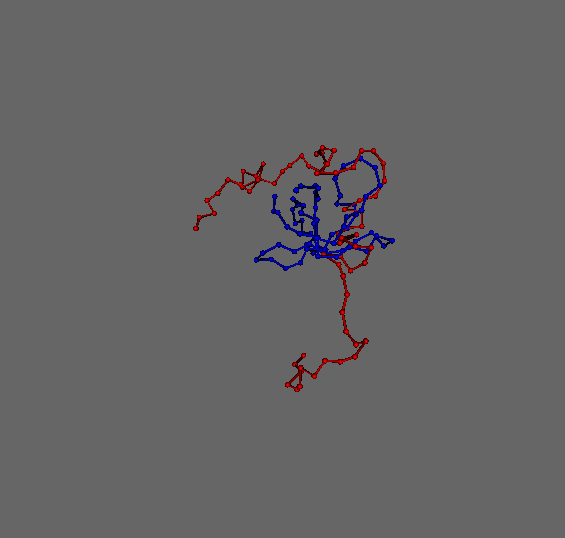

Supplement: Movie S3 — Approximate solution to minimal distance transformation from an unfolded conformation of protein 1CSP to the folded conformation, where the chains are ghost chains; instances of self-crossing are emphasized. (GIF) [file pone.0053642.s003.gif]
