# Supplementary material for: Polymer Uncrossing and Knotting in Protein Folding, and Their Role in Minimal Folding Pathways
Source: PLoS One. 2013 Jan 24;8(1):e53642. doi: 10.1371/journal.pone.0053642 (PMC3554774; doi:10.1371/journal.pone.0053642)
Supplement: File S1 — Includes: Description and analysis showing that crossing detection and uncrossing distance are independent of the projection plane, including Figure SA; Structural alignment statistics of our protein dataset, including Table SA, Table SB, and Figure SB; Cross correlation tables of order parameters, Tables SC-SJ; References. (PDF) [file pone.0053642.s004.pdf]

Polymer uncrossing and knotting in protein folding, and their role in minimal folding pathways

Ali Reza Mohazab[a] **and Steven S. Plotkin***[a]

[a] *Dept. of Physics and Astronomy, University of British Columbia, Vancouver, Canada; Tel: (604)822-8813; E-mail: steve@physics.ubc.ca*

Movie captions

Movie 1 Approximate solution to minimal distance transformation from a vertical line to a horizontal one

Movie 2 Approximate solution to minimal distance transformation from an unfolded conformation of protein 1CSP to the folded conformation, where the chains are ghost chains

Movie 3 Approximate solution to minimal distance transformation from an unfolded conformation of protein 1CSP to the folded conformation, where the chains are ghost chains; instances of self-crossing are emphasized.

Crossing detection and uncrossing distance are independent of the projection plane

Here we illustrate that the choice of projection plane does not affect the detection of a real crossing event for the protein in three-dimensional space. Consider two projections of a given configuration of cold-shock protein (1CSP), shown in figure S1 below. One projection is onto the xy plane, and another projection is onto the yz plane. At the instant shown, the protein is undergoing a crossing event in three-dimensional space between links 51 and 57, shown in blue in the figure.

The crossing matrix $\mathbb{X}$ just before and just after this instant depends on the choice of projection plane, so call the crossing matrix of each projection $\mathbb{X}_{xy}$ and $\mathbb{X}_{yz}$ respectively. The nonzero elements of $\mathbb{X}_{xy}$ are different for the xy and yz projections. However, the element (51,57) of both $\mathbb{X}_{xy}$ and $\mathbb{X}_{yz}$ will undergo a sign change at the instant of crossing (see equations 1a,b in the main text). In general, the same element of the crossing matrix $\mathbb{X}$ will change sign upon a crossing event, regardless of the projection.

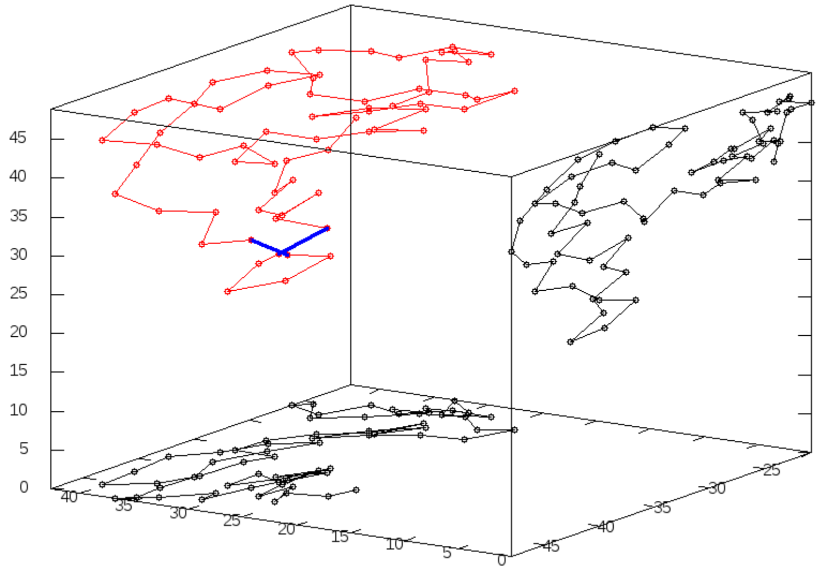

Figure S1: Two projections of a single configuration. The crossing links are shown in blue

To be specific, just before the crossing event, the crossing matrix $\mathbb{X}_{xy}$ is a sparse matrix with nonzero elements, indicated by (row, column,value), as follows:

$(1,8,-0.86), (2,8,-1.16), (3,5,-2.48), (3,7,-2.79), (3,8,-2.56), (5,3,4.72), (5,8,-4.76), (7,3,6.82), (8,1,7.55), (8,2,7.47), (8,3,7.05), (8,5,7.13), (11,13,10.26), (13,11,-12.57), (23,28,22.76), (24,26,23.47), (24,27,23.24), (26,24,-25.43), (27,24,-26.75), (28,23,-27.26), (30,39,-29.86), (31,42,-30.50), (34,43,-33.11), (39,30,38.41), (42,31,41.23), (43,34,42.97), (51,57,50.82), (52,58,-51.55), (52,60,-51.46), (53,58,-52.70), (53,60,-52.29), (54,56,-53.23), (56,54,55.67), (57,51,-56.04), (57,60,-56.39), (58,52,57.31), (58,53,57.55), (58,60,-57.34), (60,52,59.32), (60,53,59.05), (60,57,59.58), (60,58,59.25)$

Just after the crossing, the crossing matrix $\mathbb{X}_{xy}$ changes to:

$(1,8,-0.86), (2,8,-1.16), (3,5,-2.48), (3,7,-2.79), (3,8,-2.56), (5,3,4.72), (5,8,-4.76), (7,3,6.82), (8,1,7.55), (8,2,7.47), (8,3,7.05), (8,5,7.13), (11,13,10.26), (13,11,-12.57), (23,28,22.77), (24,26,23.47), (24,27,23.24), (26,24,-25.43), (27,24,-26.75), (28,23,-27.25), (30,39,-29.82), (31,42,-30.49), (34,43,-33.14), (39,30,38.43), (42,31,41.23), (43,34,42.99), (51,57,-50.97), (52,58,-51.55), (52,60,-51.43), (53,58,-52.70), (53,60,-52.30), (54,56,-53.23), (56,54,55.67), (57,51,56.16), (57,60,-56.39), (58,52,57.30), (58,53,57.55), (58,60,-57.34), (60,52,59.35), (60,53,59.05), (60,57,59.57), (60,58,59.25)$

Note that, although all elements have changed numerical values slightly (most changes are too small to be seen in the numbers above), two (conjugate) elements underwent a change of sign, namely $(51,57,50.82)$ changed to $(51,57,-50.97)$ after the crossing event, and $(57,51,-56.04)$ changed to $(57,51,56.16)$.

For the yz projection, the crossing matrix $\mathbb{X}_{yz}$ before the crossing event is given by:

$(2,16,1.06), (2,17,1.05), (5,8,-4.93), (6,8,-5.72), (8,5,7.37), (8,6,7.18), (8,10,-7.97), (10,8,9.27), (12,17,11.89), (12,18,11.78), (12,19,11.62), (14,24,13.46), (14,25,13.68), (15,17,14.96), (16,2,-15.70), (17,2,-16.09), (17,12,-16.85), (17,15,-16.32), (18,12,-17.19), (19,12,-18.32), (24,14,-23.75), (25,14,-24.06), (34,64,33.45), (37,66,36.44), (51,57,50.82), (57,51,-56.05), (64,34,-63.80), (66,37,-65.49)$

while after the transformation, $\mathbb{X}_{yz}$ changes to:

$(2,16,1.06), (2,17,1.05), (5,8,-4.93), (6,8,-5.72), (8,5,7.37), (8,6,7.18), (8,10,-7.97), (10,8,9.27), (12,17,11.89), (12,18,11.79), (12,19,11.63), (14,24,13.47), (14,25,13.70), (15,17,14.96), (16,2,-15.70), (17,2,-16.09), (17,12,-16.85), (17,15,-16.32), (18,12,-17.18), (19,12,-18.33), (24,14,-23.73), (25,14,-24.06), (34,64,33.46), (37,66,36.43), (51,57,-50.83), (57,51,56.05), (64,34,-63.80), (66,37,-65.48)$

Again the same elements underwent a change of sign: here the values are $(51,57,50.82)$ and $(57,51,-56.05)$ before the crossing event, and $(51,57,-50.83)$ and $(57,51,56.05)$ after the crossing event.

Since the uncrossing distance $\mathcal{D}_{\rm nx}$ is calculated from the links which have changed sign corresponding to crossing events, the uncrossing distance is independent of the projection used.

Structural alignment statistics

Distributions of TM-score [1] for the non-redundant (NR) database of Thiruv *et. al.* [2] (version 3), our dataset, and the proteins in references [3, 4] from which non-knotted proteins in our dataset were taken are given in Figure S2 below. By computing the overlap integral between

Table S1: Mean structural alignment scores of protein datasets (diagonal) and statistical difference tests of the alignment distributions

mean value	M-P[a]	Ivankov[b]	Gromiha[c]	NR[d]
M-P	0.306	1.1e-3	0.1680	1.2e-14
Ivankov	2.5e-4	0.295	0.018	1.2e-13
Gromiha	4.9e-3	0.13	0.302	0
NR	1.0e-11	1.2e-13	0	0.278

[a]Present analysis.

[b]Proteins from reference [3].

[c]Proteins from reference [4].

[d]Proteins from non-redundant database in reference [2].

Table S2: Structural homologues and TM-score

Protein pair		mean TM-score
1CBI	1IFC	0.855
1EAL	1IFC	0.818
1CIS	2CI2	0.820
1CSP	1MJC	0.870
1NYF	1SHG	0.843
1NS5	1O6D	0.882
1PKS	1SHG	0.824

Protein structural homologs in our Dataset

probability distributions, about 98% of the proteins in our dataset have TM-scores consistent with values in the NR database. The kinetically-based datasets, including our dataset, contain a small number of structural homologs not present in the NR database. The total list of structural homologue is given in Table S2.

The mean value of structural overlap by TM-score is given in the diagonal to Table S1. Though the mean values of TM-score are quite comparable, the sizes of the datasets are large enough for the smal differences to be signficant by the Welch T test (upper triangular part of the table). As well, a Kolmogorov-Smirnov test (lower triangular part) indicates that most of the distributions are statistically different. Our dataset is not statistically distinguishable from that of Gromiha *et. al.* by the Welsh T-test, and the datasets of Gromiha and Ivankov are indistiguishable by the Kolmogorov-Smirnov test.

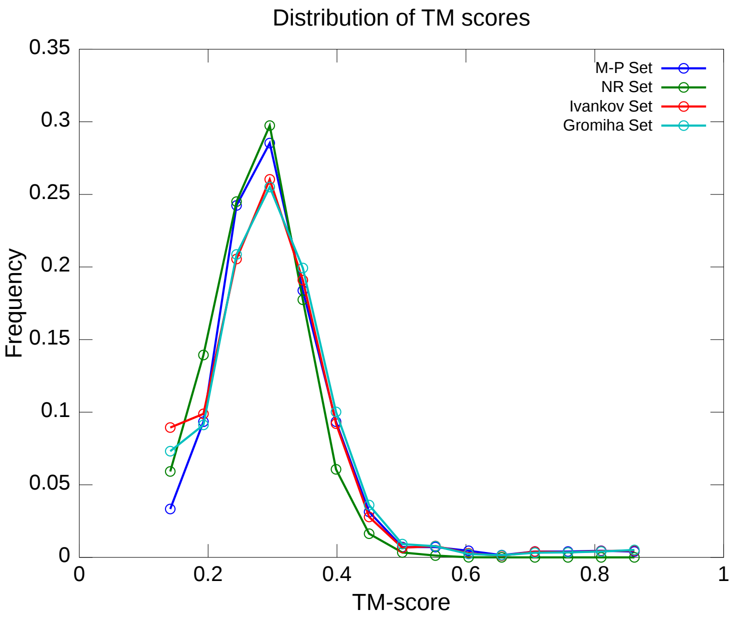

Figure S2: Distributions of TM-scores for all pairwise alignments in 4 datasets: Our dataset (M-P), the non-redundant protein dataset of [2], and those in references [3, 4].

.

Cross correlation

Cross correlation of the order parameters with each other, for various classifications of proteins are shown below. The pair (correlation coef, p-value) is deemed significant if the correlation coefficient is equal to or greater than 0.5 and p-value is smaller 0.01.

	INX	LRO	RCO	ACO	MRSD	RMSD
INX	———	(0.437,2.18e-03)	(0.413,3.78e-03)	(0.273,0.055)	(0.140,0.327)	(0.133,0.350)
LRO	0.650,4.34e-04	———	0.718,4.91e-07	0.591,3.46e-05	(0.337,0.018)	(0.357,0.012)
RCO	0.552,4.20e-03	0.853,6.25e-08	———	0.513,3.22e-04	(0.220,0.123)	(0.253,0.076)
ACO	(0.470,0.018)	0.830,2.79e-07	0.786,3.26e-06	———	0.667,3.00e-06	0.700,9.36e-07
MRSD	(0.454,0.023)	0.629,7.60e-04	(0.455,0.022)	0.867,2.09e-08	———	0.967,1.26e-11
RMSD	(0.446,0.025)	0.642,5.41e-04	(0.473,0.017)	0.880,6.77e-09	0.998,0.00e+00	———
$\langle \mathcal{D}_{\rm nx} \rangle$	0.669,2.55e-04	0.707,7.73e-05	0.546,4.73e-03	0.871,1.48e-08	0.909,3.27e-10	0.911,2.49e-10
$\langle \mathcal{D}_{\rm nx} \rangle/N$	0.918,1.07e-10	0.756,1.25e-05	0.636,6.36e-04	0.737,2.60e-05	0.737,2.61e-05	0.734,3.01e-05
$\langle \mathcal{D} \rangle$	(0.299,0.146)	0.514,8.55e-03	(0.308,0.134)	0.817,6.22e-07	0.963,1.33e-14	0.967,4.22e-15
$\langle \mathcal{D} \rangle/N$	0.534,5.93e-03	0.667,2.68e-04	(0.495,0.012)	0.877,9.00e-09	0.995,0.00e+00	0.993,0.00e+00
N	(0.213,0.306)	(0.425,0.034)	(0.160,0.444)	0.712,6.56e-05	0.917,1.22e-10	0.921,7.08e-11
	$\langle \mathcal{D}_{\rm nx} \rangle$	$\langle \mathcal{D}_{\rm nx} \rangle/N$	$\langle \mathcal{D} \rangle$	$\langle \mathcal{D} \rangle/N$	N	
INX	(0.487,6.50e-04)	0.733,2.78e-07	(0.060,0.674)	(0.207,0.148)	(0.00e+00,1.000)	
LRO	0.551,1.13e-04	0.518,2.88e-04	(0.284,0.047)	(0.377,8.20e-03)	(0.212,0.138)	
RCO	(0.487,6.50e-04)	0.533,1.86e-04	(0.153,0.283)	(0.273,0.055)	(0.081,0.573)	
ACO	0.693,1.19e-06	0.513,3.22e-04	0.627,1.13e-05	0.707,7.37e-07	0.570,6.41e-05	
MRSD	0.640,7.32e-06	(0.407,4.38e-03)	0.880,7.02e-10	0.933,6.18e-11	0.799,2.19e-08	
nRMSD	0.647,5.87e-06	(0.400,5.07e-03)	0.873,9.42e-10	0.927,8.43e-11	0.792,2.87e-08	
$\langle \mathcal{D}_{\rm nx} \rangle$	———	0.753,1.30e-07	0.573,5.89e-05	0.693,1.19e-06	0.503,4.21e-04	
$\langle \mathcal{D}_{\rm nx} \rangle/N$	0.904,6.18e-10	———	(0.327,0.022)	(0.473,9.12e-04)	(0.255,0.074)	
$\langle \mathcal{D} \rangle$	0.877,9.11e-09	0.621,9.27e-04	———	0.840,3.97e-09	0.919,1.18e-10	
$\langle \mathcal{D} \rangle/N$	0.938,4.67e-12	0.799,1.70e-06	0.946,9.69e-13	———	0.758,1.07e-07	
N	0.781,4.09e-06	0.512,8.88e-03	0.973,4.44e-16	0.889,2.81e-09	———	

Table S3: Two-state proteins: correlation between various order parameters. The upper triangle matrix (containing elements above the dash cell in each column) contains the Kendall correlation coefficient and the corresponding p-value, and the lower triangle portion contains the Pearson corr. coefficient and the corresponding p-value. The sample size is 25.

	INX	LRO	RCO	ACO	MRSD	RMSD
INX	———	(0.359,0.088)	(-2.56e-02,0.903)	(0.359,0.088)	(0.436,0.038)	(0.410,0.051)
LRO	0.714,6.13e-03	———	0.615,3.41e-03	(0.538,0.010)	(0.256,0.222)	(0.282,0.180)
RCO	(-4.88e-02,0.874)	(0.612,0.026)	———	(0.462,0.028)	(0.026,0.903)	(0.051,0.807)
ACO	(0.591,0.033)	0.703,7.31e-03	(0.516,0.071)	———	0.564,7.27e-03	0.590,5.01e-03
MRSD	0.741,3.75e-03	(0.351,0.240)	(-1.44e-01,0.639)	0.717,5.78e-03	———	0.974,3.54e-06
RMSD	0.721,5.38e-03	(0.356,0.233)	(-1.04e-01,0.736)	0.743,3.63e-03	0.997,8.57e-14	———
$\langle\mathcal{D}_{\rm nx}\rangle$	0.868,1.19e-04	(0.408,0.166)	(-3.00e-01,0.320)	(0.588,0.034)	0.898,3.13e-05	0.884,6.11e-05
$\langle\mathcal{D}_{\rm nx}\rangle/N$	0.947,9.03e-07	(0.577,0.039)	(-1.20e-01,0.696)	(0.677,0.011)	0.897,3.29e-05	0.885,5.89e-05
$\langle\mathcal{D}\rangle$	0.750,3.16e-03	(0.260,0.390)	(-3.54e-01,0.235)	(0.586,0.035)	0.955,3.70e-07	0.944,1.29e-06
$\langle\mathcal{D}\rangle/N$	0.781,1.60e-03	(0.389,0.189)	(-1.42e-01,0.643)	0.721,5.44e-03	0.998,2.38e-14	0.994,8.54e-12
N	0.733,4.36e-03	(0.204,0.505)	(-4.61e-01,0.113)	(0.485,0.093)	0.905,2.16e-05	0.884,5.96e-05
	$\langle\mathcal{D}_{\rm nx}\rangle$	$\langle\mathcal{D}_{\rm nx}\rangle/N$	$\langle\mathcal{D}\rangle$	$\langle\mathcal{D}\rangle/N$	N	
INX	0.615,3.41e-03	0.769,2.52e-04	(0.513,0.015)	(0.436,0.038)	(0.503,0.017)	
LRO	(0.333,0.113)	(0.333,0.113)	(0.231,0.272)	(0.256,0.222)	(0.219,0.297)	
RCO	(-5.13e-02,0.807)	(-5.13e-02,0.807)	(-5.13e-02,0.807)	(0.026,0.903)	(-9.03e-02,0.667)	
ACO	(0.487,0.020)	(0.436,0.038)	(0.487,0.020)	0.564,7.27e-03	(0.452,0.032)	
MRSD	0.769,2.52e-04	0.667,1.51e-03	0.821,9.44e-05	1.000,1.95e-06	0.735,4.65e-04	
RMSD	0.795,1.55e-04	0.641,2.29e-03	0.846,5.66e-05	0.974,3.54e-06	0.761,2.91e-04	
$\langle\mathcal{D}_{\rm nx}\rangle$	———	0.846,5.66e-05	0.897,1.95e-05	0.769,2.52e-04	0.890,2.27e-05	
$\langle\mathcal{D}_{\rm nx}\rangle/N$	0.966,8.34e-08	———	0.744,4.02e-04	0.667,1.51e-03	0.735,4.65e-04	
$\langle\mathcal{D}\rangle$	0.959,2.42e-07	0.899,2.97e-05	———	0.821,9.44e-05	0.916,1.30e-05	
$\langle\mathcal{D}\rangle/N$	0.920,8.61e-06	0.924,6.46e-06	0.959,2.17e-07	———	0.735,4.65e-04	
N	0.934,2.93e-06	0.857,1.83e-04	0.983,1.78e-09	0.909,1.64e-05	———	

Table S4: Three-state proteins: correlation between various order parameters. The upper triangle matrix (containing elements above the dash cell in each column) contains the Kendall correlation coefficient and the corresponding p-value, and the lower triangle portion contains the Pearson corr. coefficient and the corresponding p-value. The sample size is 13.

	INX	LRO	RCO	ACO	MRSD	RMSD
INX	———	(0.330,0.157)	(-3.09e-01,0.186)	(0.345,0.139)	(0.491,0.036)	(0.418,0.073)
LRO	(0.372,0.259)	———	(0.183,0.432)	0.624,7.56e-03	0.624,7.56e-03	(0.550,0.018)
RCO	(-4.71e-01,0.144)	(0.208,0.538)	———	(0.200,0.392)	(-9.09e-02,0.697)	(-1.82e-02,0.938)
ACO	(0.381,0.248)	0.753,7.53e-03	(0.172,0.613)	———	0.709,2.40e-03	0.782,8.15e-04
MRSD	(0.586,0.058)	(0.600,0.051)	(-2.04e-01,0.547)	0.910,9.88e-05	———	0.927,7.18e-05
RMSD	(0.560,0.073)	(0.599,0.051)	(-1.94e-01,0.567)	0.918,6.68e-05	0.999,7.84e-13	———
$\langle \mathcal{D}_{\rm nx} \rangle$	(0.600,0.051)	(0.425,0.193)	(-3.60e-01,0.277)	0.786,4.12e-03	0.928,3.77e-05	0.927,3.96e-05
$\langle \mathcal{D}_{\rm nx} \rangle /N$	0.852,8.71e-04	(0.494,0.123)	(-4.22e-01,0.196)	(0.723,0.012)	0.898,1.72e-04	0.886,2.80e-04
$\langle \mathcal{D} \rangle$	(0.510,0.109)	(0.490,0.126)	(-2.78e-01,0.409)	0.858,7.22e-04	0.964,1.72e-06	0.967,1.17e-06
$\langle \mathcal{D} \rangle /N$	(0.616,0.044)	(0.595,0.053)	(-2.26e-01,0.504)	0.901,1.53e-04	0.999,7.26e-14	0.997,3.38e-11
N	(0.535,0.090)	(0.578,0.062)	(-2.65e-01,0.431)	0.897,1.80e-04	0.984,4.42e-08	0.988,1.14e-08
	$\langle \mathcal{D}_{\rm nx} \rangle$	$\langle \mathcal{D}_{\rm nx} \rangle /N$	$\langle \mathcal{D} \rangle$	$\langle \mathcal{D} \rangle /N$	N	
INX	0.673,3.97e-03	0.818,4.60e-04	(0.491,0.036)	(0.527,0.024)	(0.587,0.012)	
LRO	(0.587,0.012)	(0.440,0.059)	0.624,7.56e-03	(0.587,0.012)	0.611,8.88e-03	
RCO	(-1.27e-01,0.586)	(-2.00e-01,0.392)	(-9.09e-02,0.697)	(-1.27e-01,0.586)	(-1.10e-01,0.637)	
ACO	0.673,3.97e-03	(0.527,0.024)	0.709,2.40e-03	0.673,3.97e-03	0.697,2.83e-03	
MRSD	0.818,4.60e-04	0.673,3.97e-03	1.000,1.85e-05	0.964,3.69e-05	0.917,8.55e-05	
RMSD	0.745,1.41e-03	(0.600,0.010)	0.927,7.18e-05	0.891,1.36e-04	0.844,3.01e-04	
$\langle \mathcal{D}_{\rm nx} \rangle$	———	0.855,2.53e-04	0.818,4.60e-04	0.855,2.53e-04	0.844,3.01e-04	
$\langle \mathcal{D}_{\rm nx} \rangle /N$	0.925,4.64e-05	———	0.673,3.97e-03	0.709,2.40e-03	0.697,2.83e-03	
$\langle \mathcal{D} \rangle$	0.983,5.84e-08	0.882,3.24e-04	———	0.964,3.69e-05	0.917,8.55e-05	
$\langle \mathcal{D} \rangle /N$	0.936,2.31e-05	0.915,7.76e-05	0.965,1.58e-06	———	0.881,1.62e-04	
N	0.948,9.44e-06	0.881,3.36e-04	0.982,7.80e-08	0.983,5.91e-08	———	

Table S5: α-helix dominated proteins (both 2- and 3- state): Correlation between various order parameters. The upper triangle matrix (containing elements above the dash cell in each column) contains the Kendall correlation coefficient and the corresponding p-value, and the lower triangle portion contains the Pearson corr. coefficient and the corresponding p-value. The sample size is 11.

	INX	LRO	RCO	ACO	MRSD	RMSD
INX	———	(0.165,0.412)	(0.363,0.071)	(-2.75e-01,0.171)	(-2.97e-01,0.139)	(-3.85e-01,0.055)
LRO	(0.035,0.904)	———	0.626,1.81e-03	(0.165,0.412)	(-3.30e-02,0.870)	(-7.69e-02,0.702)
RCO	(0.378,0.183)	0.676,7.92e-03	———	(-7.69e-02,0.702)	(-3.63e-01,0.071)	(-3.19e-01,0.112)
ACO	(-1.86e-01,0.524)	(0.342,0.231)	(-2.82e-01,0.328)	———	0.714,3.73e-04	0.758,1.58e-04
MRSD	(-2.91e-01,0.313)	(-1.84e-01,0.529)	(-7.61e-01,1.57e-03)	0.826,2.75e-04	———	0.912,5.52e-06
RMSD	(-3.09e-01,0.283)	(-1.90e-01,0.516)	(-7.52e-01,1.92e-03)	0.830,2.37e-04	0.998,1.55e-15	———
$\langle \mathcal{D}_{\rm nx} \rangle$	(-9.97e-02,0.734)	(-2.13e-01,0.465)	(-7.26e-01,3.26e-03)	0.812,4.18e-04	0.978,1.73e-09	0.972,5.98e-09
$\langle \mathcal{D}_{\rm nx} \rangle/N$	(0.328,0.252)	(-1.35e-01,0.644)	(-5.09e-01,0.063)	0.707,4.68e-03	0.807,4.89e-04	0.794,7.03e-04
$\langle \mathcal{D} \rangle$	(-2.46e-01,0.396)	(-2.30e-01,0.429)	(-7.68e-01,1.33e-03)	0.812,4.24e-04	0.993,1.16e-12	0.991,7.58e-12
$\langle \mathcal{D} \rangle/N$	(-2.44e-01,0.400)	(-1.83e-01,0.532)	(-7.52e-01,1.94e-03)	0.828,2.55e-04	0.999,0.00e+00	0.996,9.30e-14
N	(-2.80e-01,0.332)	(-1.69e-01,0.564)	(-7.43e-01,2.32e-03)	0.844,1.47e-04	0.994,1.05e-12	0.992,2.89e-12
	$\langle \mathcal{D}_{\rm nx} \rangle$	$\langle \mathcal{D}_{\rm nx} \rangle/N$	$\langle \mathcal{D} \rangle$	$\langle \mathcal{D} \rangle/N$	N	
INX	(-9.89e-02,0.622)	(0.187,0.352)	(-3.63e-01,0.071)	(-2.97e-01,0.139)	(-3.76e-01,0.061)	
LRO	(0.077,0.702)	(-7.69e-02,0.702)	(-1.10e-02,0.956)	(-3.30e-02,0.870)	(-2.21e-02,0.912)	
RCO	(-1.21e-01,0.547)	(-9.89e-02,0.622)	(-2.97e-01,0.139)	(-3.63e-01,0.071)	(-2.87e-01,0.152)	
ACO	0.648,1.24e-03	(0.363,0.071)	0.780,1.02e-04	0.714,3.73e-04	0.796,7.39e-05	
MRSD	0.758,1.58e-04	(0.516,0.010)	0.934,3.27e-06	1.000,6.30e-07	0.928,3.76e-06	
RMSD	0.714,3.73e-04	(0.429,0.033)	0.934,3.27e-06	0.912,5.52e-06	0.950,2.20e-06	
$\langle \mathcal{D}_{\rm nx} \rangle$	———	0.714,3.73e-04	0.736,2.45e-04	0.758,1.58e-04	0.729,2.80e-04	
$\langle \mathcal{D}_{\rm nx} \rangle/N$	0.900,1.14e-05	———	(0.451,0.025)	(0.516,0.010)	(0.442,0.028)	
$\langle \mathcal{D} \rangle$	0.988,3.69e-11	0.824,2.90e-04	———	0.934,3.27e-06	0.994,7.26e-07	
$\langle \mathcal{D} \rangle/N$	0.986,1.20e-10	0.835,2.04e-04	0.994,6.02e-13	———	0.928,3.76e-06	
N	0.981,6.64e-10	0.805,5.11e-04	0.996,2.98e-14	0.993,2.25e-12	———	

Table S6: β-sheet dominated proteins (both 2- and 3- state): Correlation between various order parameters. The upper triangle matrix (containing elements above the dash cell in each column) contains the Kendall correlation coefficient and the corresponding p-value, and the lower triangle portion contains the Pearson corr. coefficient and the corresponding p-value. The sample size is 14.

	INX	LRO	RCO	ACO	MRSD	RMSD
INX	———	(0.077,0.714)	(-3.59e-01,0.088)	(0.128,0.542)	(0.385,0.067)	(0.359,0.088)
LRO	(0.308,0.307)	———	(0.308,0.143)	(0.487,0.020)	(0.282,0.180)	(0.308,0.143)
RCO	(-4.26e-01,0.147)	(0.521,0.068)	———	(0.205,0.329)	(-2.05e-01,0.329)	(-1.79e-01,0.393)
ACO	(0.300,0.320)	(0.664,0.013)	(0.463,0.111)	———	0.590,5.01e-03	0.615,3.41e-03
MRSD	(0.670,0.012)	(0.340,0.255)	(-2.30e-01,0.451)	0.726,4.95e-03	———	0.974,3.54e-06
RMSD	(0.659,0.014)	(0.373,0.209)	(-1.89e-01,0.537)	0.758,2.66e-03	0.998,8.22e-15	———
$\langle\mathcal{D}_{\rm nx}\rangle$	0.751,3.09e-03	(0.217,0.475)	(-5.13e-01,0.073)	(0.481,0.096)	0.915,1.14e-05	0.909,1.70e-05
$\langle\mathcal{D}_{\rm nx}\rangle/N$	0.889,4.79e-05	(0.328,0.275)	(-3.90e-01,0.187)	(0.545,0.054)	0.921,7.87e-06	0.916,1.11e-05
$\langle\mathcal{D}\rangle$	(0.683,0.010)	(0.212,0.487)	(-4.83e-01,0.095)	(0.534,0.060)	0.940,1.85e-06	0.932,3.41e-06
$\langle\mathcal{D}\rangle/N$	0.709,6.61e-03	(0.342,0.253)	(-2.56e-01,0.399)	0.706,6.94e-03	0.998,5.55e-15	0.996,6.81e-13
N	(0.668,0.013)	(0.143,0.641)	(-5.76e-01,0.039)	(0.437,0.136)	0.897,3.28e-05	0.882,6.66e-05

	$\langle\mathcal{D}_{\rm nx}\rangle$	$\langle\mathcal{D}_{\rm nx}\rangle/N$	$\langle\mathcal{D}\rangle$	$\langle\mathcal{D}\rangle/N$	N	
INX	0.590,5.01e-03	0.590,5.01e-03	(0.513,0.015)	(0.410,0.051)	(0.462,0.028)	
LRO	(0.179,0.393)	(0.179,0.393)	(0.205,0.329)	(0.308,0.143)	(0.154,0.464)	
RCO	(-3.08e-01,0.143)	(-3.08e-01,0.143)	(-2.82e-01,0.180)	(-1.79e-01,0.393)	(-2.82e-01,0.180)	
ACO	(0.436,0.038)	(0.436,0.038)	(0.513,0.015)	0.615,3.41e-03	(0.513,0.015)	
MRSD	0.795,1.55e-04	0.795,1.55e-04	0.872,3.35e-05	0.974,3.54e-06	0.821,9.44e-05	
RMSD	0.769,2.52e-04	0.769,2.52e-04	0.846,5.66e-05	0.949,6.34e-06	0.795,1.55e-04	
$\langle\mathcal{D}_{\rm nx}\rangle$	———	0.949,6.34e-06	0.923,1.12e-05	0.821,9.44e-05	0.872,3.35e-05	
$\langle\mathcal{D}_{\rm nx}\rangle/N$	0.948,8.33e-07	———	0.872,3.35e-05	0.821,9.44e-05	0.821,9.44e-05	
$\langle\mathcal{D}\rangle$	0.986,6.07e-10	0.915,1.14e-05	———	0.897,1.95e-05	0.949,6.34e-06	
$\langle\mathcal{D}\rangle/N$	0.929,4.31e-06	0.942,1.50e-06	0.946,1.07e-06	———	0.846,5.66e-05	
N	0.960,1.93e-07	0.874,9.26e-05	0.985,8.98e-10	0.902,2.43e-05	———	

Table S7: Mixed secondary structure proteins: Correlation between various parameters. The upper triangle matrix (containing elements above the dash cell in each column) contains the Kendall correlation coefficient and the corresponding p-value, and the lower triangle portion contains the Pearson corr. coefficient and the corresponding p-value. The sample size is 13.

	INX	LRO	RCO	ACO	MRSD	RMSD
INX	———	(0.424,1.77e-04)	(0.229,0.043)	(0.292,9.96e-03)	(0.252,0.026)	(0.223,0.048)
LRO	0.658,7.03e-06	———	0.615,5.48e-08	0.518,4.66e-06	(0.262,0.021)	(0.268,0.018)
RCO	(0.297,0.071)	0.736,1.42e-07	———	(0.340,2.66e-03)	(4.27e-03,0.970)	(0.033,0.772)
ACO	0.516,9.10e-04	0.730,1.98e-07	(0.494,1.61e-03)	———	0.642,1.43e-08	0.670,3.19e-09
MRSD	0.513,1.00e-03	(0.403,0.012)	(-4.60e-02,0.784)	0.805,1.07e-09	———	0.954,0.00e+00
RMSD	0.502,1.33e-03	(0.411,0.010)	(-2.62e-02,0.876)	0.819,3.32e-10	0.998,0.00e+00	———
$\langle \mathcal{D}_{\rm nx} \rangle$	0.591,9.27e-05	(0.344,0.034)	(-1.83e-01,0.271)	0.672,3.83e-06	0.901,1.31e-14	0.895,3.60e-14
$\langle \mathcal{D}_{\rm nx} \rangle/N$	0.856,7.02e-12	0.587,1.07e-04	(0.109,0.514)	0.745,8.28e-08	0.851,1.27e-11	0.844,2.69e-11
$\langle \mathcal{D} \rangle$	(0.432,6.78e-03)	(0.241,0.145)	(-2.63e-01,0.111)	0.673,3.68e-06	0.949,0.00e+00	0.945,0.00e+00
$\langle \mathcal{D} \rangle/N$	0.566,2.12e-04	(0.434,6.48e-03)	(-2.65e-02,0.874)	0.811,6.52e-10	0.998,0.00e+00	0.995,0.00e+00
N	(0.395,0.014)	(0.211,0.203)	(-3.26e-01,0.046)	0.627,2.56e-05	0.934,0.00e+00	0.928,0.00e+00
	$\langle \mathcal{D}_{\rm nx} \rangle$	$\langle \mathcal{D}_{\rm nx} \rangle/N$	$\langle \mathcal{D} \rangle$	$\langle \mathcal{D} \rangle/N$	N	
INX	(0.488,1.62e-05)	0.633,2.21e-08	(0.218,0.054)	(0.289,0.011)	(0.180,0.111)	
LRO	(0.379,8.18e-04)	(0.381,7.47e-04)	(0.211,0.063)	(0.276,0.015)	(0.167,0.139)	
RCO	(0.115,0.309)	(0.147,0.195)	(-5.83e-02,0.606)	(0.024,0.831)	(-1.03e-01,0.363)	
ACO	0.633,2.21e-08	0.545,1.47e-06	0.596,1.38e-07	0.656,6.81e-09	0.560,7.31e-07	
MRSD	0.747,4.10e-11	0.619,4.53e-08	0.886,4.88e-15	0.963,0.00e+00	0.826,2.82e-13	
RMSD	0.724,1.56e-10	0.590,1.82e-07	0.881,7.11e-15	0.929,2.22e-16	0.823,3.40e-13	
$\langle \mathcal{D}_{\rm nx} \rangle$	———	0.849,6.13e-14	0.730,1.12e-10	0.778,6.12e-12	0.689,1.13e-09	
$\langle \mathcal{D}_{\rm nx} \rangle/N$	0.907,4.22e-15	———	0.579,3.11e-07	0.656,6.81e-09	0.538,2.03e-06	
$\langle \mathcal{D} \rangle$	0.961,0.00e+00	0.814,5.13e-10	———	0.866,1.91e-14	0.941,0.00e+00	
$\langle \mathcal{D} \rangle/N$	0.917,6.66e-16	0.885,1.61e-13	0.947,0.00e+00	———	0.806,1.03e-12	
N	0.928,0.00e+00	0.769,1.74e-08	0.987,0.00e+00	0.928,0.00e+00	———	

Table S8: Unknotted proteins: correlation between various order parameters the upper triangle matrix (containing elements above the dash cell in each column) contains the Kendall correlation coefficient and the corresponding p-value, and the lower triangle portion contains the Pearson corr. coefficient and the corresponding p-value. The sample size is 38.

	INX	LRO	RCO	ACO	MRSD	RMSD
INX	———	(0.524,0.099)	(-5.24e-01,0.099)	(-1.43e-01,0.652)	(-2.38e-01,0.453)	(-1.43e-01,0.652)
LRO	(0.767,0.044)	———	(-4.76e-02,0.881)	(-2.38e-01,0.453)	(-3.33e-01,0.293)	(-4.29e-01,0.176)
RCO	(-7.22e-01,0.067)	(-3.26e-01,0.476)	———	(-1.43e-01,0.652)	(-2.38e-01,0.453)	(-3.33e-01,0.293)
ACO	(-6.24e-02,0.894)	(-3.92e-01,0.385)	(-3.94e-01,0.382)	———	0.905,4.32e-03	(0.810,0.011)
MRSD	(0.213,0.647)	(-1.15e-01,0.805)	(-7.13e-01,0.072)	0.901,5.68e-03	———	0.905,4.32e-03
RMSD	(0.211,0.649)	(-1.33e-01,0.776)	(-7.19e-01,0.068)	0.900,5.77e-03	0.999,1.27e-08	———
$\langle \mathcal{D}_{\mathrm{nx}} \rangle$	(0.713,0.072)	(0.304,0.508)	(-9.73e-01,2.29e-04)	(0.530,0.221)	(0.789,0.035)	(0.792,0.034)
$\langle \mathcal{D}_{\mathrm{nx}} \rangle/N$	0.919,3.42e-03	(0.602,0.153)	(-9.05e-01,5.09e-03)	(0.287,0.533)	(0.573,0.179)	(0.571,0.180)
$\langle \mathcal{D} \rangle$	(0.344,0.450)	(-8.12e-02,0.863)	(-8.31e-01,0.021)	(0.832,0.020)	0.970,2.84e-04	0.975,1.92e-04
$\langle \mathcal{D} \rangle/N$	(0.396,0.379)	(0.044,0.926)	(-8.16e-01,0.025)	(0.830,0.021)	0.981,9.09e-05	0.981,1.00e-04
N	(0.365,0.421)	(-8.59e-02,0.855)	(-8.41e-01,0.018)	(0.822,0.023)	0.957,7.22e-04	0.962,5.15e-04
	$\langle \mathcal{D}_{\mathrm{nx}} \rangle$	$\langle \mathcal{D}_{\mathrm{nx}} \rangle/N$	$\langle \mathcal{D} \rangle$	$\langle \mathcal{D} \rangle/N$	N	
INX	(0.714,0.024)	0.905,4.32e-03	(0.143,0.652)	(0.048,0.881)	(0.238,0.453)	
LRO	(0.238,0.453)	(0.429,0.176)	(-3.33e-01,0.293)	(-2.38e-01,0.453)	(-2.38e-01,0.453)	
RCO	(-8.10e-01,0.011)	(-6.19e-01,0.051)	(-6.19e-01,0.051)	(-5.24e-01,0.099)	(-7.14e-01,0.024)	
ACO	(0.143,0.652)	(-4.76e-02,0.881)	(0.524,0.099)	(0.619,0.051)	(0.429,0.176)	
MRSD	(0.048,0.881)	(-1.43e-01,0.652)	(0.619,0.051)	(0.714,0.024)	(0.524,0.099)	
RMSD	(0.143,0.652)	(-4.76e-02,0.881)	(0.714,0.024)	(0.810,0.011)	(0.619,0.051)	
$\langle \mathcal{D}_{\mathrm{nx}} \rangle$	———	(0.810,0.011)	(0.429,0.176)	(0.333,0.293)	(0.524,0.099)	
$\langle \mathcal{D}_{\mathrm{nx}} \rangle/N$	0.924,2.97e-03	———	(0.238,0.453)	(0.143,0.652)	(0.333,0.293)	
$\langle \mathcal{D} \rangle$	0.890,7.33e-03	(0.678,0.094)	———	(0.714,0.024)	0.905,4.32e-03	
$\langle \mathcal{D} \rangle/N$	0.885,8.07e-03	(0.720,0.068)	0.981,9.40e-05	———	(0.619,0.051)	
N	0.899,5.90e-03	(0.690,0.086)	0.998,2.16e-07	0.972,2.37e-04	———	

Table S9: Knotted proteins: correlating between various order parameters the upper triangle matrix (containing elements above the dash cell in each column) contains the Kendall correlation coefficient and the corresponding p-value, and the lower triangle portion contains the Pearson corr. coefficient and the corresponding p-value. The sample size is 7.

	INX	LRO	RCO	ACO	MRSD	RMSD
INX	———	(0.361,4.76e-04)	(0.048,0.639)	(0.352,6.63e-04)	(0.380,2.35e-04)	(0.358,5.34e-04)
LRO	0.530,1.81e-04	———	(0.488,2.28e-06)	(0.448,1.45e-05)	(0.217,0.035)	(0.223,0.031)
RCO	(0.022,0.883)	0.662,7.48e-07	———	(0.200,0.053)	(-1.23e-01,0.233)	(-1.05e-01,0.309)
ACO	0.548,9.85e-05	0.619,5.95e-06	(0.334,0.025)	———	0.661,1.58e-10	0.679,4.91e-11
MRSD	0.637,2.55e-06	(0.322,0.031)	(-1.82e-01,0.232)	0.832,1.47e-12	———	0.962,0.00e+00
RMSD	0.625,4.48e-06	(0.329,0.027)	(-1.64e-01,0.282)	0.843,3.57e-13	0.999,0.00e+00	———
$\langle\mathcal{D}_{\mathrm{nx}}\rangle$	0.776,3.85e-10	(0.238,0.115)	(-3.31e-01,0.027)	0.659,8.70e-07	0.875,4.00e-15	0.868,1.13e-14
$\langle\mathcal{D}_{\mathrm{nx}}\rangle/N$	0.919,0.00e+00	(0.419,4.21e-03)	(-1.45e-01,0.344)	0.698,9.91e-08	0.851,1.34e-13	0.842,4.14e-13
$\langle\mathcal{D}\rangle$	0.622,5.13e-06	(0.174,0.254)	(-3.68e-01,0.013)	0.722,2.17e-08	0.954,0.00e+00	0.951,0.00e+00
$\langle\mathcal{D}\rangle/N$	0.697,1.07e-07	(0.345,0.020)	(-1.79e-01,0.239)	0.827,2.54e-12	0.996,0.00e+00	0.994,0.00e+00
N	0.583,2.66e-05	(0.160,0.293)	(-4.13e-01,4.78e-03)	0.693,1.31e-07	0.947,0.00e+00	0.944,0.00e+00
	$\langle\mathcal{D}_{\mathrm{nx}}\rangle$	$\langle\mathcal{D}_{\mathrm{nx}}\rangle/N$	$\langle\mathcal{D}\rangle$	$\langle\mathcal{D}\rangle/N$	N	
INX	0.592,9.90e-09	0.707,7.51e-12	(0.370,3.43e-04)	(0.420,4.71e-05)	(0.329,1.42e-03)	
LRO	(0.324,1.68e-03)	(0.318,2.05e-03)	(0.183,0.076)	(0.233,0.024)	(0.146,0.157)	
RCO	(-5.66e-02,0.584)	(-3.43e-02,0.739)	(-1.74e-01,0.092)	(-1.11e-01,0.282)	(-2.20e-01,0.033)	
ACO	0.634,8.08e-10	0.556,7.44e-08	0.622,1.68e-09	0.669,9.43e-11	0.583,1.65e-08	
MRSD	0.776,5.77e-14	0.673,7.27e-11	0.889,0.00e+00	0.960,0.00e+00	0.832,8.88e-16	
RMSD	0.758,2.19e-13	0.651,2.98e-10	0.887,0.00e+00	0.933,0.00e+00	0.832,8.88e-16	
$\langle\mathcal{D}_{\mathrm{nx}}\rangle$	———	0.877,0.00e+00	0.778,4.97e-14	0.812,3.55e-15	0.735,1.10e-12	
$\langle\mathcal{D}_{\mathrm{nx}}\rangle/N$	0.953,0.00e+00	———	0.655,2.31e-10	0.713,4.97e-12	0.611,3.23e-09	
$\langle\mathcal{D}\rangle$	0.947,0.00e+00	0.862,2.69e-14	———	0.885,0.00e+00	0.940,0.00e+00	
$\langle\mathcal{D}\rangle/N$	0.906,0.00e+00	0.893,2.22e-16	0.959,0.00e+00	———	0.824,1.33e-15	
N	0.916,0.00e+00	0.823,3.78e-12	0.990,0.00e+00	0.947,0.00e+00	———	

Table S10: All proteins: correlating between various order parameters the upper triangle matrix (containing elements above the dash cell in each column) contains the Kendall correlation coefficient and the corresponding p-value, and the lower triangle portion contains the Pearson corr. coefficient and the corresponding p-value. The sample size is 45.

References

[1] Zhang Y, Skolnick J (2005) Tm-align: a protein structure alignment algorithm based on the tm-score. Nucleic Acids Research 33: 2302-2309.

[2] Thiruv B, Quon G, Saldanha S, Steipe B (2005) Nh3d: A reference dataset of non-homologous protein structures. BMC structural biology 5: 12.

[3] Ivankov DN, Garbuzynskiy SO, Alm E, Plaxco KW, Baker D, et al. (2003) Contact order revisited: Influence of protein size on the folding rate. Protein Science 12: 2057–2062.

[4] Gromiha M, Thangakani A, Selvaraj S (2006) Fold-rate: prediction of protein folding rates from amino acid sequence. Nucleic acids research 34: W70–W74.
